# Myofibroblast YAP/TAZ activation is a key step in organ fibrogenesis

**DOI:** 10.1172/jci.insight.146243

**Published:** 2022-02-22

**Authors:** Xiaolin He, Monica F. Tolosa, Tianzhou Zhang, Santosh Kumar Goru, Luisa Ulloa Severino, Paraish S. Misra, Caitríona M. McEvoy, Lauren Caldwell, Stephen G. Szeto, Feng Gao, Xiaolan Chen, Cassandra Atin, Victoria Ki, Noah Vukosa, Catherine Hu, Johnny Zhang, Christopher Yip, Adriana Krizova, Jeffrey L. Wrana, Darren A. Yuen

**Affiliations:** 1 Keenan Research Centre for Biomedical Science, Li Ka Shing Knowledge Institute, St. Michael’s Hospital (Unity Health Toronto) and Department of Medicine, and; 2 Center for Systems Biology, Lunenfeld-Tanenbaum Research Institute, Mt. Sinai Hospital and Department of Molecular Genetics, University of Toronto, Toronto, Ontario, Canada.; 3 Department of Pathology, The Third Hospital of Hebei Medical University, Shijiazhuang, Hebei, People’s Republic of China.; 4 Department of Respiratory and Critical Care Medicine, Beijing Shijitan Hospital, Capital Medical University, Beijing, People’s Republic of China.; 5 Faculty of Applied Science and Engineering, University of Toronto, Toronto, Ontario, Canada.; 6 Department of Laboratory Medicine and Pathobiology, St. Michael’s Hospital (Unity Health Toronto) and University of Toronto, Toronto, Ontario, Canada.

**Keywords:** Nephrology, Pulmonology, Chronic kidney disease, Fibrosis

## Abstract

Fibrotic diseases account for nearly half of all deaths in the developed world. Despite its importance, the pathogenesis of fibrosis remains poorly understood. Recently, the two mechanosensitive transcription cofactors YAP and TAZ have emerged as important profibrotic regulators in multiple murine tissues. Despite this growing recognition, a number of important questions remain unanswered, including which cell types require YAP/TAZ activation for fibrosis to occur and the time course of this activation. Here, we present a detailed analysis of the role that myofibroblast YAP and TAZ play in organ fibrosis and the kinetics of their activation. Using analyses of cells, as well as multiple murine and human tissues, we demonstrated that myofibroblast YAP and TAZ were activated early after organ injury and that this activation was sustained. We further demonstrated the critical importance of myofibroblast YAP/TAZ in driving progressive scarring in the kidney, lung, and liver, using multiple transgenic models in which YAP and TAZ were either deleted or hyperactivated. Taken together, these data establish the importance of early injury-induced myofibroblast YAP and TAZ activation as a key event driving fibrosis in multiple organs. This information should help guide the development of new antifibrotic YAP/TAZ inhibition strategies.

## Introduction

Fibrosis is a final common pathway of injury that underlies the pathogenesis of tissue damage in many chronic diseases (1). Thought to be primarily mediated by scar-producing cells called myofibroblasts, fibrosis leads to replacement of healthy parenchymal cells with pathological extracellular matrix, a process that not only reduces organ function but also leads to capillary loss and further tissue damage (2, 3). Reflecting its important role as a driver of chronic organ injury, fibrosis is estimated to account for nearly half of all deaths in the developed world (1). Despite its critical role in driving chronic tissue damage and dysfunction, few safe and effective antifibrotic therapies exist, sparking intense interest in better understanding the signaling pathways that contribute to fibrogenesis.

Despite the myriad insults that can occur across different tissues, a set of conserved profibrotic pathways is largely responsible for the scarring that ensues (1, 4). Recent reports have implicated the closely related transcription cofactors Yes-associated protein (YAP) and transcriptional coactivator with PDZ-binding motif (TAZ) as critical drivers of the fibrogenic response (5–15). YAP/TAZ signaling is activated by multiple cues, including TGF-β (16) and tissue stiffening (17–19), both of which are important features of fibrosing organs (20, 21). Working independently and via interactions with other profibrotic pathways, such as TGF-β (7, 22–24), EGFR (13, 14), MRTF (25), and Wnt signaling (12), YAP and TAZ play important roles as coordinators of profibrotic signaling.

Although the role of YAP and TAZ as profibrotic molecules has been established, a number of key questions remain unanswered. Firstly, the specific cell type(s) responsible for YAP/TAZ-mediated fibrogenesis remain(s) nebulous because many of the initial reports documenting the importance of YAP and TAZ used systemically administered pharmacological inhibitors (6, 15, 24). Although several follow-up conditional transgenic mouse studies have since been performed, none have definitively examined the importance of myofibroblasts in mediating the profibrotic effects of YAP and TAZ in multiple organs (9, 14). Secondly, the kinetics of YAP/TAZ activation after organ injury have not been well studied, and yet this information is necessary to guide the timing of YAP/TAZ inhibitor initiation as a potential antifibrotic strategy.

Here, we examined the role of myofibroblast YAP/TAZ activation in organ fibrogenesis. Using mouse models of kidney, lung, and liver fibrosis, as well as human biopsy samples, we demonstrated that in myofibroblasts, YAP/TAZ activation was a key step that occurred early after injury in multiple organs in mice and humans. We further demonstrated that myofibroblast-specific deficiency of LATS1 and LATS2, the Hippo pathway kinases that inhibit YAP/TAZ, strongly augmented myofibroblast YAP/TAZ activation after organ injury and exacerbated fibrosis. In contrast, myofibroblast-specific deletion of YAP/TAZ reduced scarring. Taken together, our data established a critical and conserved role for myofibroblast Hippo pathway inactivation (and subsequent YAP/TAZ activation) in driving organ fibrosis.

## Results

### Human organ fibrosis is associated with YAP and TAZ activation.

Fibrosis is a prominent histopathological finding in the setting of chronic kidney transplant injury in humans (2–4, 26). Thus, to explore whether YAP and TAZ are activated after human organ injury, we examined a set of 18 archival human transplant kidney biopsies that had undergone bulk RNA-Seq (unpublished observations). These 18 samples consisted mostly of clinically indicated biopsies performed for new onset allograft dysfunction, as well as several protocol biopsies collected as part of an independent, prospective cohort study (see Methods for more details). Our cohort consisted of 12 males and 6 females, with the mean age being 51 ± 12 years, and the biopsies being performed on average 4.4 ± 3.3 years after transplant. The serum creatinine at the time of the biopsy was 165 ± 52 μmol/L, and the calculated estimated glomerular filtration rate (eGFR) was 39 ± 13 mL/min/1.73 m^2^.

**Figure 1:**
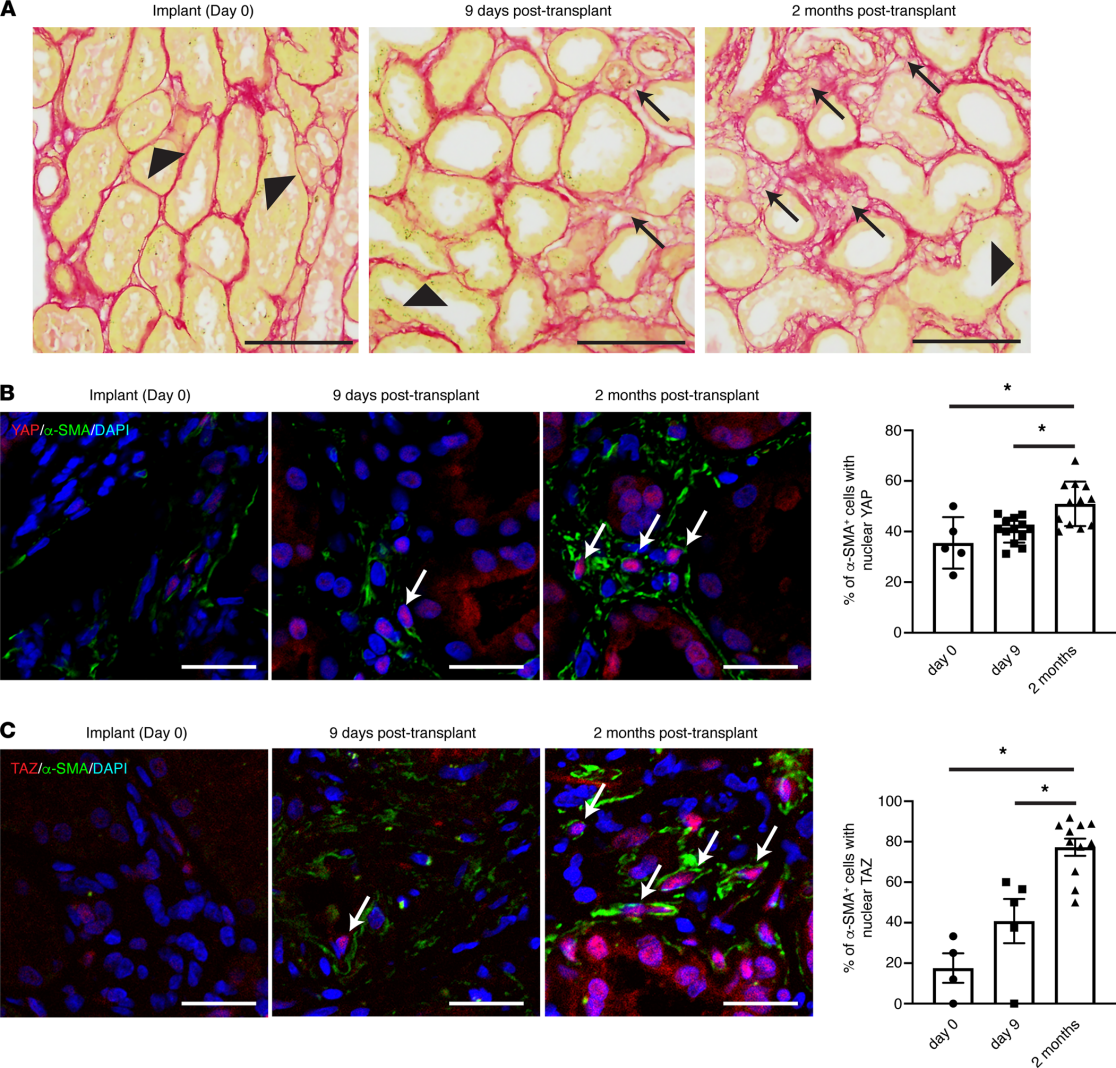
Progressive myofibroblast YAP/TAZ activation occurs during human kidney fibrogenesis. (**A**) Serial biopsies collected from the same renal allograft at different times intratransplant and after transplant revealed a progressive increase in interstitial fibrosis, as evidenced by increased picrosirius red staining over time. Black arrows point to increased deposition of collagen (pathologic matrix), whereas black arrowheads identify tubular basement membrane. Black scale bar: 100 μm. (**B**) Kidney sections were costained with antibodies directed against α-smooth muscle actin (α-SMA, green) and YAP (red) with DAPI (blue) nuclear counterstaining. Each dot in the graph represents a single image (*n* = 5 images day 0, *n* = 13 images day 9, and *n* = 12 images month 2). White arrows point to α-SMA^+^ myofibroblasts with predominantly nuclear YAP staining. White scale bar: 25 μm. The percentage of α-SMA^+^ myofibroblasts with predominantly nuclear YAP staining was quantified. (**C**) Kidney sections were similarly analyzed for nuclear TAZ localization in α-SMA^+^ myofibroblasts (α-SMA: green, TAZ: red, DAPI: blue). Each dot in the graph represents a single image (*n* = 4 images day 0, *n* = 5 images day 9, and *n* = 11 images month 2). White scale bar: 25 μm. One-way ANOVA with post hoc Tukey’s test was used for comparisons. Data shown as mean ± SEM. *P < 0.05.

Of the 18 biopsies analyzed, 9 had minimal-mild interstitial fibrosis (ci [interstitial fibrosis] 0–1) and 9 had moderate-severe fibrosis (ci 2–3), as determined by a blinded pathologist review. We found that a YAP-associated transcriptional signature (27) was enriched in the moderate-severe fibrosis group (YAP gene set variation analysis [GSVA] enrichment score: 0.13 ± 0.05 for moderate-severe fibrosis vs. –0.13 ± 0.11 for minimal-mild fibrosis, *P* < 0.05, 2-tailed Student’s *t* test), suggesting that YAP activation may be a feature of fibrosing kidneys. We next stained a set of archival kidney transplant biopsies obtained serially from a transplant recipient whose allograft demonstrated increasing fibrotic burden over time (Figure 1). Consistent with our RNA-Seq findings, we noted a progressive increase in nuclear YAP and TAZ staining (a marker of YAP/TAZ activation) in α-smooth muscle actin–positive (SMA^+^) fibroblasts (Figure 1). Taken together, our data suggests that fibroblast YAP and TAZ are activated in the fibrosing human kidney after injury.

To determine whether this finding is restricted to the kidney or is more representative of human organ fibrosis in general, we next examined a recently published single-cell RNA-Seq data set derived from nonfibrotic and fibrotic human lungs (28). In line with our findings in human transplant kidneys, the YAP-associated transcriptional signature was also increased in fibroblasts isolated from fibrotic versus nonfibrotic human lungs (adjusted *P* = 2.8 × 10^–7^).

### Fibrosis is associated with YAP/TAZ activation in multiple organs.

**Figure 2:**
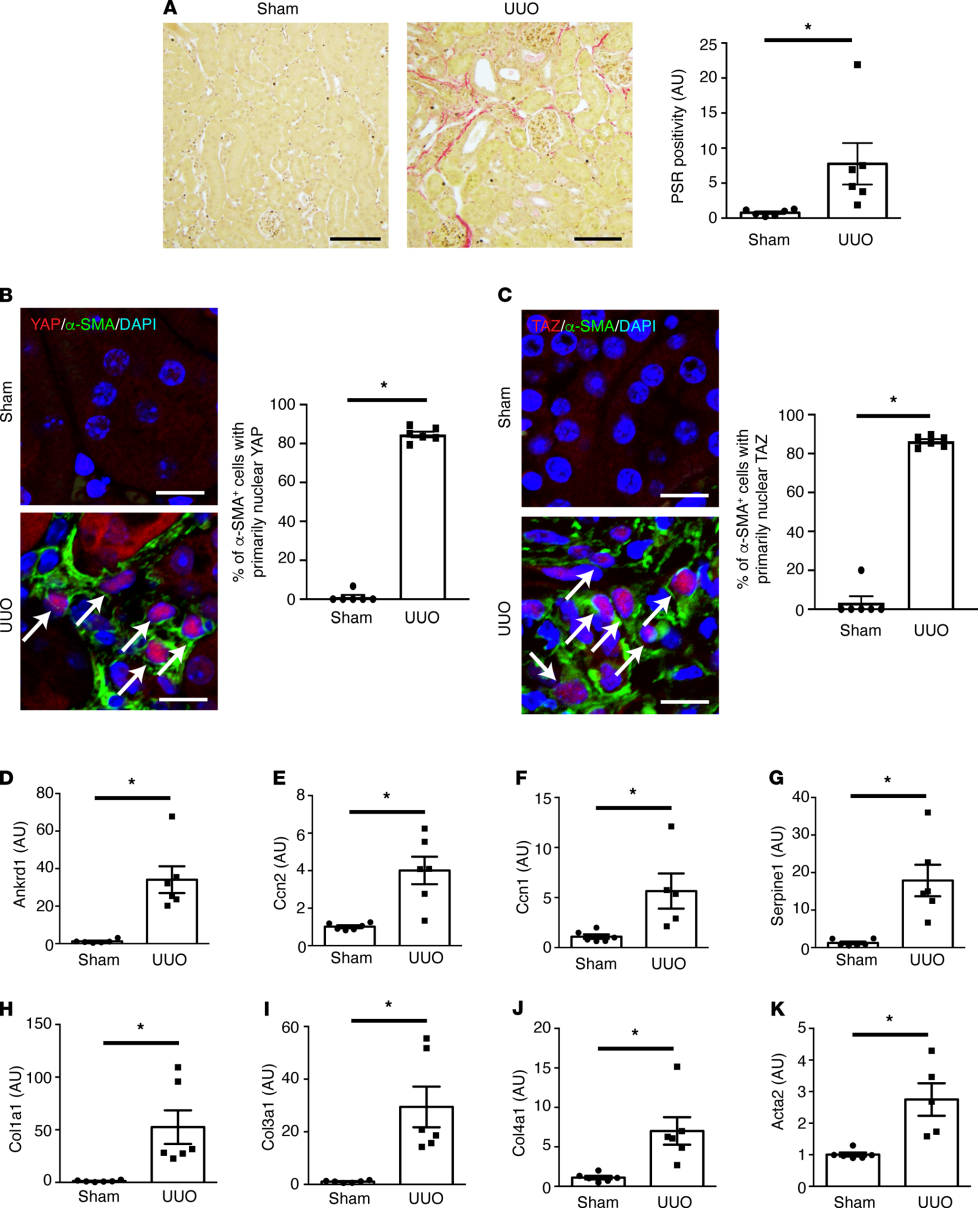
Activation of myofibroblast YAP and TAZ after renal fibrotic injury. Male C57BL/6 mice underwent sham (*n* = 6) or left-sided unilateral ureteral obstruction (UUO, *n* = 6) surgery. Mice were euthanized 7 days after surgery. (**A**) Kidney sections were stained with picrosirius red (PSR) to quantify fibrillar collagen (*n* = 6 kidneys/group). Black scale bar: 100 μm. (**B**) To quantify myofibroblast YAP activation, kidney sections were costained with antibodies targeting α-smooth muscle actin (α-SMA, green) and YAP (red) with nuclear DAPI counterstaining (blue). White scale bar: 10 μm (**C**) Similar staining was done to detect myofibroblast TAZ activation (α-SMA: green, TAZ: red, DAPI: blue). White arrows depict green α-SMA^+^ cells that have predominant red YAP or TAZ nuclear staining. White scale bar: 10 μm. The mRNA levels of the YAP/TAZ-inducible genes (**D**) *Ankrd1*, (**E**) *Ccn2*, (**F**) *Ccn1*, and (**G**) *Serpine1*, as well as those of the fibrosis-associated genes (**H**) *Col1a1*, (**I**) *Col3a1*, (**J**) *Col4a1*, and (**K**) *Acta2* were examined using qPCR of cDNA prepared from whole kidney homogenates. Transcript levels were normalized to the housekeeper transcript *Gapdh*. A 2-tailed Student’s *t* test was used for comparisons. Data shown as mean ± SEM. *P < 0.05.

We next turned to well-established mouse models of organ fibrosis to dissect this fibroblast YAP and TAZ activation event in more detail. We first examined mice undergoing left-sided unilateral ureteral obstruction (UUO), a model of kidney fibrosis (29). Seven days after UUO surgery, the left kidney demonstrated significant fibrosis (Figure 2A and Supplemental Figure 1; supplemental material available online with this article; https://doi.org/10.1172/jci.insight.146243DS1). We noted that α-SMA^+^ myofibroblasts demonstrated increased YAP and TAZ nuclear localization, indicating that fibrotic injury was associated with myofibroblast YAP/TAZ activation in this model (Figure 2, B and C). Consistent with our histological findings, we also demonstrated increased expression of YAP/TAZ target (Figure 2, D–G) and fibrosis-associated genes (Figure 2, H–K). We found similar results in mice with bleomycin-induced lung fibrosis (Supplemental Figure 2).

Finally, we analyzed 3 publicly available single-cell RNA-Seq data sets, 1 from murine lungs after bleomycin injection (30), and 2 from murine livers damaged with CCl_4_ (31, 32). Consistent with our findings, lung fibroblasts isolated 21 days after bleomycin injection (when fibrosis is present) demonstrated an increased YAP transcriptional signature compared with lung fibroblasts isolated from uninjured mice (Supplemental Figure 3A). Similarly, portal fibroblasts and hepatic stellate cells isolated from CCl_4_-induced fibrotic livers expressed higher levels of YAP-regulated genes compared with their counterparts isolated from control livers (Supplemental Figure 3, B–D).

### YAP/TAZ are activated in myofibroblasts early after injury.

**Figure 3:**
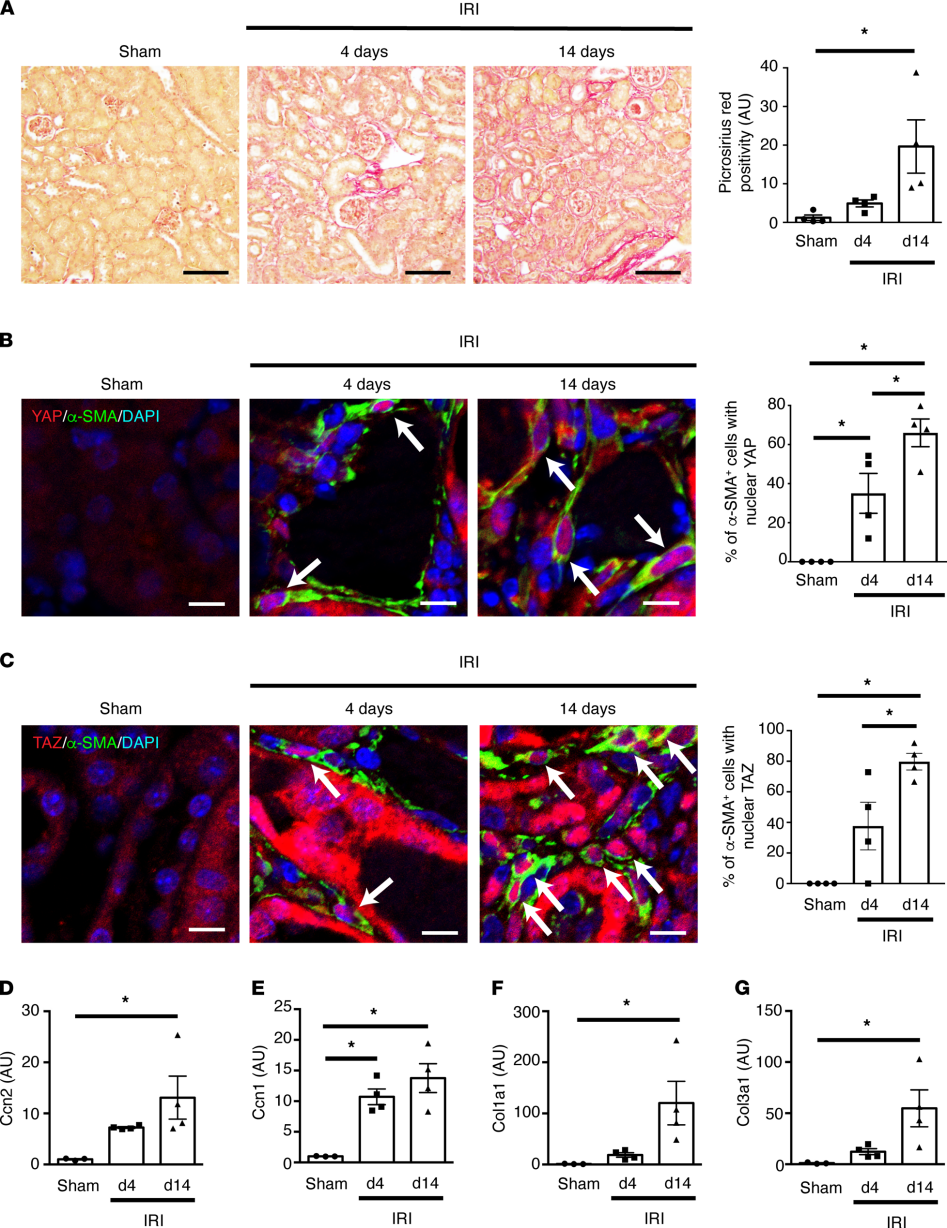
Myofibroblast YAP and TAZ are progressively activated during renal fibrogenesis. Male C57BL/6 mice underwent sham (*n* = 4) or left-sided unilateral ischemia/reperfusion injury (IRI, *n* = 8) surgery. IRI mice were euthanized at early (4 days, *n* = 4) or late (14 days, *n* = 4) time points after injury. Sham-operated mice were euthanized at 14 days after surgery. (**A**) Kidney sections were stained with picrosirius red to quantify fibrotic injury. Black scale bar: 100 μm. (**B**) To quantify myofibroblast YAP activation, kidneys were costained with antibodies directed against α-smooth muscle actin (α-SMA, green) and YAP (red) with nuclear DAPI counterstaining (blue). White scale bar: 10 μm. (**C**) Myofibroblast TAZ activation was similarly assessed after costaining for α-SMA (green), TAZ (red), and DAPI (blue). White arrows depict green α-SMA^+^ cells that have predominantly red YAP or TAZ nuclear staining. White scale bar: 10 μm. (**D–G**) The mRNA levels of the YAP/TAZ-inducible genes (**D**) *Ccn2* and (**E**) *Ccn1* and the fibrosis-associated genes (**F**) *Col1a1* and (**G**) *Col3a1* were examined using qPCR of cDNA prepared from whole kidney homogenates. Transcript levels were normalized to the housekeeper transcript *Gapdh*. One-way ANOVA with post hoc Tukey’s test was used for comparisons. Data shown as mean ± SEM. *P < 0.05. d4, 4 days after-IRI. d14, 14 days after IRI.

To assess when YAP/TAZ activation occurs after injury, we next examined mouse kidneys at various time points after disease onset. For this purpose, we subjected mice to unilateral left-sided ischemia/reperfusion injury (IRI), a model of progressive fibrosis in which ischemic acute kidney injury leads to fibrotic chronic kidney disease (Figure 3A) (29, 33). Immunofluorescence staining with antibodies directed against α-SMA, YAP, and TAZ demonstrated an increase in nuclear YAP and TAZ localization in α-SMA^+^ myofibroblasts by day 4 after IRI, when fibrosis has just begun to appear, suggesting that myofibroblast YAP/TAZ activation is an early event after kidney injury (Figure 3, A–C). Given that cellular stiffening is a key driver of YAP/TAZ activation (17–19), we next examined the stiffness of renal interstitial α-SMA^+^ myofibroblasts using atomic force microscopy. Consistent with our finding of increasing myofibroblast YAP/TAZ activation after IRI, we noted a progressive increase in interstitial α-SMA^+^ cell stiffness over the course of the 14-day experiment (Supplemental Figure 4). Because myofibroblasts are derived from a number of interstitial α-SMA^–^ cell populations, including pericytes and quiescent α-SMA^–^ fibroblasts (34, 35), we also measured the stiffness of α-SMA^–^ cells in the renal interstitium. Similar to our observations in α-SMA^+^ cells, we noted a progressive stiffening of these interstitial α-SMA^–^ cells as well (Supplemental Figure 4). Finally, we examined downstream expression of the YAP/TAZ-inducible genes *Ccn2* and *Ccn1* and found an increase in transcript levels at 4 days, with a further rise by 14 days after IRI (Figure 3, D and E). A similar pattern was noted when the expression of collagen transcripts was examined (Figure 3, F and G).

To confirm our findings, we examined a publicly available bulk RNA-Seq data set in which mouse kidneys were sampled at multiple time points after IRI, ranging from 2 hours to 12 months after injury (33). Consistent with our own findings, the same YAP transcriptional signature we found to be elevated in other fibrotic tissues (Supplemental Figure 3) was also increased early after IRI in mouse kidneys and remained elevated at 28 days after injury (Supplemental Figure 5) (33).

### Myofibroblast YAP/TAZ deficiency attenuates organ fibrosis.

**Figure 4:**
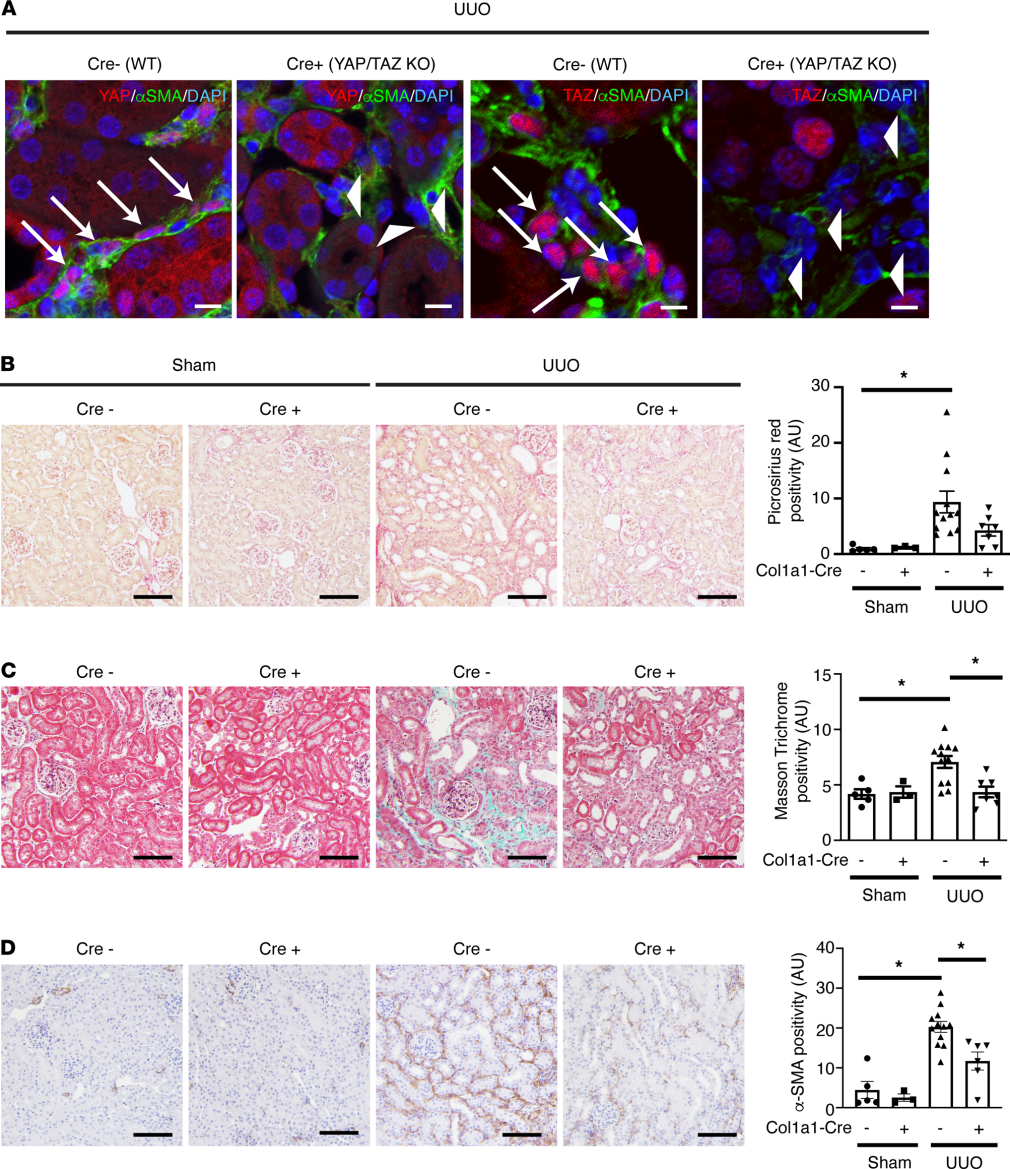
Myofibroblast-specific YAP/TAZ deficiency attenuates unilateral ureteral obstruction–induced kidney fibrosis. Myofibroblast-specific YAP/TAZ-deficient mice (Col1a1-Cre/ERT^+/–^ *Yap^fl/fl^ Taz^fl/fl^*) and their WT littermates (Col1a1-Cre/ERT^–/–^ *Yap^fl/fl^* *Taz^fl/fl^*) were randomized to sham surgery (*n* = 3 Col1a1-Cre/ERT^+/–^ *Yap^fl/fl^* *Taz^fl/fl^* and *n* = 5 Col1a1-Cre/ERT^–/–^ *Yap^fl/fl^* *Taz^fl/fl^*) or left-sided unilateral ureteral obstruction (*n* = 12 Col1a1-Cre/ERT^+/–^ *Yap^fl/fl^* *Taz^fl/fl^* and *n* = 7 Col1a1-Cre/ERT^–/–^ *Yap^fl/fl^* *Taz^fl/fl^*). Tamoxifen was administered between days 0 and 6 after surgery to activate expressed Cre recombinase. Left kidneys were harvested 7 days after surgery. (**A**) Kidney sections were stained with antibodies directed against α-smooth muscle actin (α-SMA), YAP, or TAZ, and nuclei were counterstained with DAPI to assess for successful YAP and TAZ excision in myofibroblasts. White arrows depict α-SMA^+^ cells expressing YAP. White arrowheads depict α-SMA^+^ cells without significant YAP expression. Note the lack of red YAP and TAZ staining in myofibroblasts of YAP/TAZ-KO animals (Col1a1-Cre/ERT^+/–^ *Yap^fl/fl^* *Taz^fl/fl^*). White scale bar: 10 μm. Kidney sections were next stained with (**B**) picrosirius red (PSR) to label fibrillar collagen, (**C**) Masson’s trichrome to stain extracellular matrix, or (**D**) an antibody directed against α-SMA. Black scale bar: 100 μm. One-way ANOVA with post hoc Tukey’s test was used for comparisons. Data shown as mean ± SEM. *P < 0.05.

**Figure 5:**
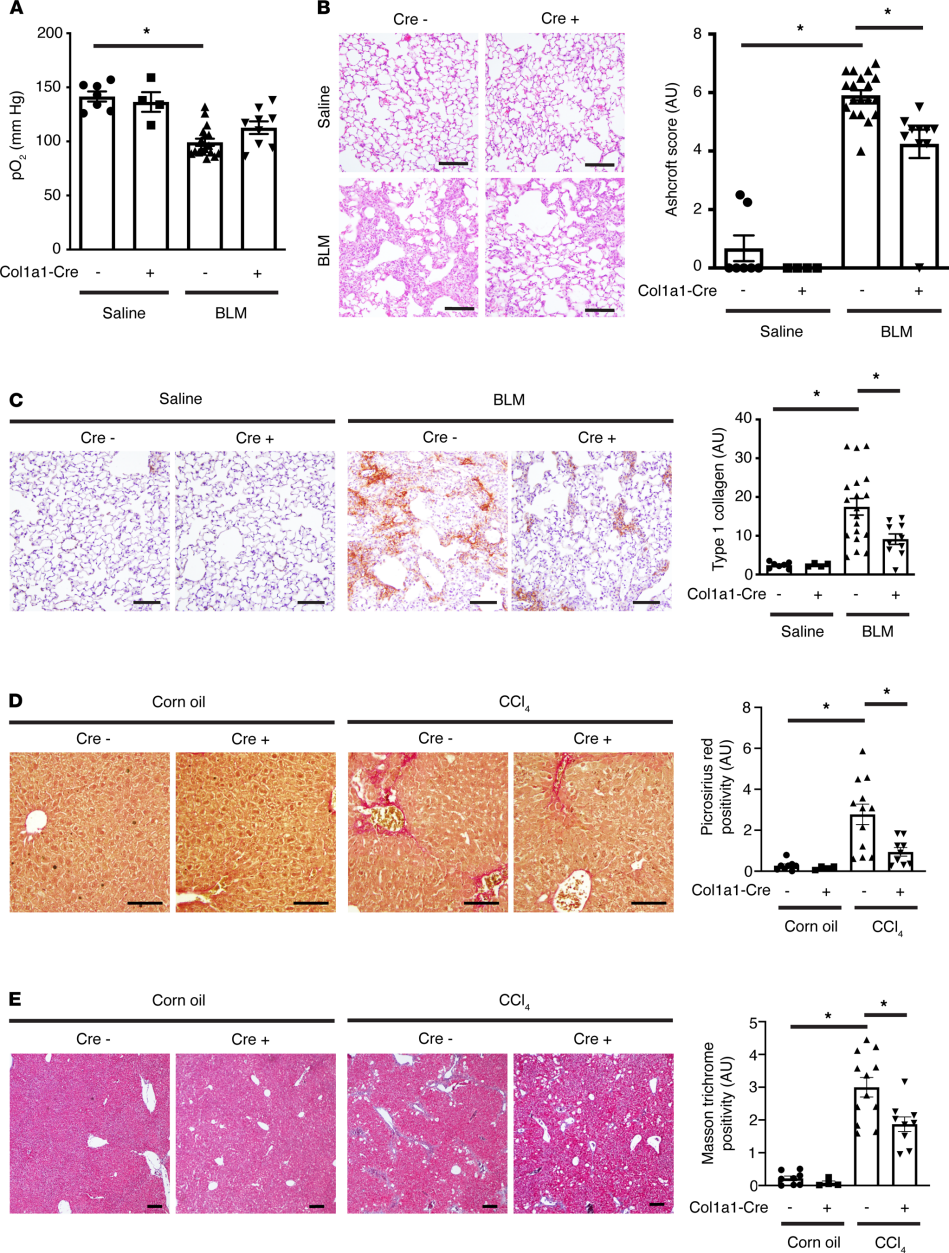
Myofibroblast-specific YAP/TAZ deficiency attenuates bleomycin-induced lung and CCl_4_-induced liver fibrosis. Myofibroblast-specific YAP/TAZ-deficient mice (Col1a1-Cre/ERT^+/–^ *Yap^fl/fl^* *Taz^fl/fl^*) and their WT littermates (Col1a1-Cre/ERT^–/–^ *Yap^fl/fl^* *Taz^fl/fl^*) were randomized to saline or bleomycin injections (*n* = 7 saline Col1a1-Cre/ERT^–/–^ *Yap^fl/fl^* *Taz^fl/fl^*, *n* = 4 saline Col1a1-Cre/ERT^+/–^ *Yap^fl/fl^* *Taz^fl/fl^*, *n* = 19 bleomycin Col1a1-Cre/ERT^–/–^ *Yap^fl/fl^* *Taz^fl/fl^*, *n* = 10 bleomycin Col1a1-Cre/ERT^+/–^ *Yap^fl/fl^* *Taz^fl/fl^*). Tamoxifen was administered between days 7 and 13 after saline/bleomycin injection, and lungs were harvested on day 14. (**A**) Arterial blood pO_2_ levels just prior to euthanization. Lung sections were stained with (**B**) H&E for Ashcroft injury scoring (a measure of lung fibrosis), or (**C**) an antibody directed against type 1 collagen. Scale bar: 100 μm. (**D** and **E**) Myofibroblast-specific YAP/TAZ-deficient mice (Col1a1-Cre/ERT^+/–^ *Yap^fl/fl^* *Taz^fl/fl^*) and their WT littermates (Col1a1-Cre/ERT^–/–^ *Yap^fl/fl^* *Taz^fl/fl^*) were randomized to corn oil (*n* = 8 Col1a1-Cre/ERT^–/–^ *Yap^fl/fl^* *Taz^fl/fl^* and *n* = 4 Col1a1-Cre/ERT^+/–^ *Yap^fl/fl^* *Taz^fl/fl^*) or CCl_4_ injections (*n* = 12 Col1a1-Cre/ERT^–/–^ *Yap^fl/fl^* *Taz^fl/fl^* and *n* = 9 Col1a1-Cre/ERT^+/–^ *Yap^fl/fl^* *Taz^fl/fl^*). Tamoxifen was administered between days 8 and 12 after the initiation of corn oil/CCl_4_ injections to activate expressed Cre recombinase. Livers were harvested 6 weeks after the first corn oil/CCl_4_ injection. Liver sections were stained with (**D**) picrosirius red (PSR) to label fibrillar collagen or (**E**) Masson’s trichrome to stain extracellular matrix. Scale bar: 100 μm. One-way ANOVA with post hoc Tukey’s test was used for comparisons. Data shown as mean ± SEM. *P < 0.05. pO_2_, partial pressure of oxygen; BLM, bleomycin.

To examine the significance of myofibroblast YAP/TAZ activation after organ injury, we next generated *Yap^fl/fl^* *Taz^fl/fl^* mice expressing a tamoxifen-inducible Cre recombinase under the control of the mouse type 1 (α1) collagen (Col1a1) promoter. These mice were subjected to UUO-induced kidney fibrosis (Figure 4), bleomycin-induced lung fibrosis (Figure 5, A–C), and CCl_4_-induced liver fibrosis (Figure 5, D and E). The timing of organ injury and tamoxifen administration for each model is summarized in Supplemental Figure 6. Tamoxifen was administered either during (UUO and bleomycin) or at the beginning (CCl_4_) of the fibrogenic period after each type of injury (between days 0 and 6 after UUO, between days 7 and 13 after bleomycin, and between days 8 and 12 after CCl_4_) (36, 37). In all 3 models, myofibroblast-specific YAP/TAZ deficiency was associated with a reduction in organ fibrosis (Figures 4 and 5 and Supplemental Figure 8). Furthermore, in bleomycin-injured mice, knockdown of YAP and TAZ resulted in improved lung function, as evidenced by an increase in arterial blood oxygenation (Figure 5A).

Finally, we confirmed the above phenotypes with a second Cre driver, using *Yap^fl/fl^* *Taz^fl/fl^* mice expressing a tamoxifen-inducible Cre recombinase under the control of the mouse type 1 (α2) collagen (Col1a2) promoter (Supplemental Figure 7A). Col1a2-Cre/ERT^+/–^ *Yap^fl/fl^* *Taz^fl/fl^* mice again demonstrated a marked protection against UUO-induced fibrotic injury (Supplemental Figure 7).

### Overactivation of fibroblast YAP/TAZ exacerbates fibrotic injury.

**Figure 6:**
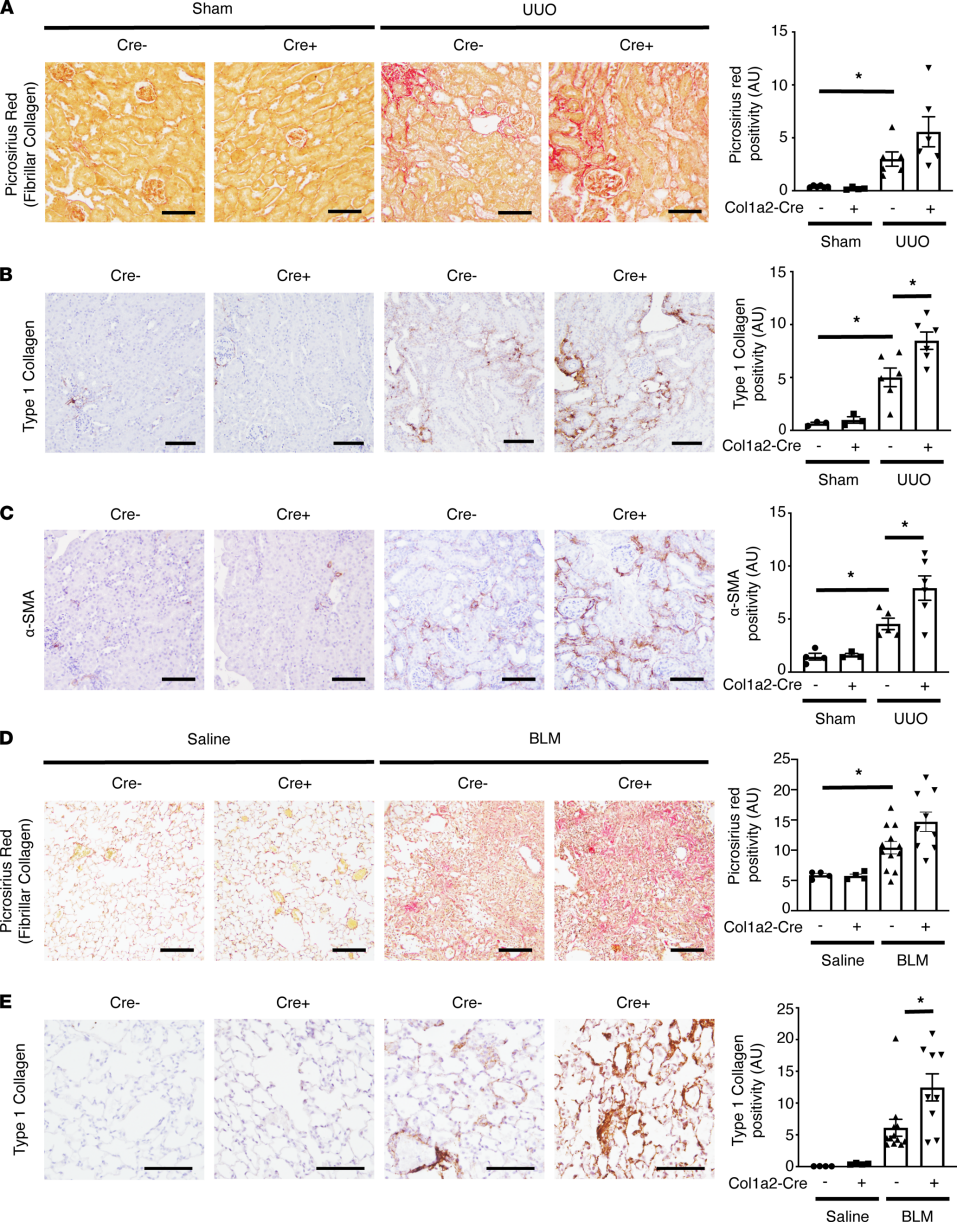
Myofibroblast-specific LATS1/2 deficiency augments renal and lung fibrosis. Myofibroblast-specific LATS1/2-deficient mice (Col1a2-Cre/ERT^+/–^ *Lats1^fl/fl^* *Lats2^fl/fl^*) and their WT littermates (Col1a2-Cre/ERT^–/–^ *Lats1^fl/fl^ Lats2^fl/fl^*) were randomized to sham surgery (*n* = 6 Col1a2-Cre/ERT^–/–^ *Lats1^fl/fl^ Lats2^fl/fl^* and *n* = 4 Col1a2-Cre/ERT^+/–^ *Lats1^fl/fl^ Lats2^fl/fl^*) or left-sided unilateral ureteral obstruction (UUO, *n* = 6 Col1a2-Cre/ERT^–/–^ *Lats1^fl/fl^ Lats2^fl/fl^* and *n* = 6 Col1a2-Cre/ERT^+/–^ *Lats1^fl/fl^ Lats2^fl/fl^*). Tamoxifen was administered between days 0 and 6 after surgery to activate expressed Cre recombinase. Left kidneys were harvested 7 days after surgery. Kidney sections were also stained with (**A**) picrosirius red (PSR) to label fibrillar collagen and antibodies directed against (**B**) type 1 collagen or (**C**) α-smooth muscle actin. (**D** and **E**) Myofibroblast-specific LATS1/2-deficient mice (Col1a2-Cre/ERT^+/–^ *Lats1^fl/fl^ Lats2^fl/fl^*) and their WT littermates (Col1a2-Cre/ERT^–/–^ *Lats1^fl/fl^ Lats2^fl/fl^*) were randomized to saline or bleomycin injections (*n* = 4 saline Col1a2-Cre/ERT^–/–^ *Lats1^fl/fl^ Lats2^fl/fl^*, *n* = 4 saline Col1a2-Cre/ERT^+/–^ *Lats1^fl/fl^ Lats2^fl/fl^*, *n* = 12 bleomycin Col1a2-Cre/ERT^–/–^ *Lats1^fl/fl^ Lats2^fl/fl^*, *n* = 9, bleomycin Col1a2-Cre/ERT^+/–^ *Lats1^fl/fl^ Lats2^fl/fl^*). Tamoxifen was administered between days 7 and 13 after saline/bleomycin injection, and lungs harvested on day 14. Lung sections were stained with (**D**) PSR to label fibrillar collagen or (**E**) an antibody directed against type 1 collagen. Scale bar: 100 μm. One-way ANOVA with post hoc Tukey’s test was used for comparisons. Data shown as mean ± SEM. *P < 0.05. BLM, bleomycin.

A key mechanism controlling YAP and TAZ activity is phosphorylation by the inhibitory Hippo pathway kinases LATS1 and LATS2 (38). We therefore tested whether deficiency of LATS1/2 in myofibroblasts would further exacerbate fibrotic injury by generating Col1a2-Cre/ERT^+/–^ *Lats1^fl/fl^ Lats2^fl/fl^* mice and subjecting them to both UUO-induced kidney or bleomycin-induced lung injury. Details of the experimental design are again summarized in Supplemental Figure 6. Myofibroblast-specific LATS1/2 deficiency led to a significant upregulation of YAP/TAZ activity, as demonstrated by an increase in YAP and TAZ nuclear localization (Supplemental Figures 9 and 10) and augmented expression of YAP/TAZ-inducible genes (Supplemental Figure 9, C and D). As expected, this increase in myofibroblast YAP/TAZ activation was associated with increased pathological matrix deposition in both the obstructed kidney (Figure 6, A–C, and Supplemental Figure 9, E–G) and the bleomycin-injured lung (Figure 6, D and E). Taken together, our data suggest that myofibroblast YAP and TAZ, and their upstream Hippo pathway regulators, are important modulators of organ fibrosis.

## Discussion

YAP and TAZ, the downstream effectors of the Hippo pathway, have emerged as critical drivers of organ fibrosis. To date, however, the specific cell types that require YAP/TAZ activation for scarring to occur have remained unclear. Here, we demonstrated that YAP and TAZ were activated in myofibroblasts early after organ injury in mice and humans. We corroborated findings in our mouse and human samples with analyses of publicly available RNA-Seq data sets in mouse and human organs with a particular focus on myofibroblasts. Next, using transgenic mice in which YAP and TAZ were either deleted or hyperactivated in a myofibroblast-specific manner, we demonstrated that this early activation of myofibroblast YAP/TAZ activity is a critical event driving scarring in multiple organs. Taken together, our results establish the importance of the myofibroblast Hippo pathway and its effectors YAP and TAZ in mediating fibrogenesis after tissue injury.

Emerging evidence suggests that YAP and/or TAZ contribute to injury-induced organ damage by promoting inflammation (39) and fibrosis (5, 6, 8, 11, 27, 40–42). In particular, a growing body of evidence has shown that YAP and/or TAZ can activate myofibroblast matrix production through TGF-β–dependent and –independent mechanisms of action (5–10, 22, 23). In particular, several groups have used transgenic approaches to demonstrate that stromal cell YAP and/or TAZ appear to play critical roles in mediating fibrosis of the kidney and heart (9, 43, 44). Our findings are broadly in line with these reports, as we showed that myofibroblast YAP/TAZ deficiency protected against injury-induced fibrosis of the kidney, lung, and liver, whereas myofibroblast YAP/TAZ overactivation induced by LATS1/2 deficiency exacerbated injury-induced scarring. Interestingly, Xiao et al. found that YAP/TAZ hyperactivation in cardiac fibroblasts induced by *Lats1/2* knockout resulted in cardiac scarring even in uninjured mice (44), a finding that we did not observe in the kidney or lung when we knocked out *Lats1/2*. Many potential explanations for this discrepancy exist, including differences in the genetic targeting strategy used and the timing of tissue analysis after *Lats1/2* knockout. However, it is also possible that the regulation of fibrogenesis, and thus the importance of (myo)fibroblast YAP and/or TAZ, may be tissue and even context specific. Clearly, future studies are required to better understand this complex process.

The biology of fibrogenesis has remained unclear, in part because the transgenic strategies used to target matrix production have largely focused on precursor cells that, after injury, differentiate into myofibroblasts, the dominant cells responsible for matrix production. Unfortunately, no single marker can be used to identify relevant precursor cell populations in different organs. Thus, choosing the myofibroblast precursor population to target when aiming to delete genes of interest is critical because it may influence the results obtained. Indeed, as alluded to above, differences in the targeting strategy used may account for differences observed between our study and others (44, 45). Our goal was to understand the role of myofibroblast YAP and TAZ in the regulation of fibrotic responses across multiple organs, so we chose type 1 collagen promoter–driven Cre transgenic mouse lines, reasoning that type 1 collagen is predominantly expressed in myofibroblasts after organ injury. Although type 1 collagen is expressed by a variety of other cell types to a lesser degree (46), using this targeting strategy, we were able to focus as much as possible on the downstream effector cells (myofibroblasts) to avoid missing any relevant precursor cell populations that contribute to myofibroblast generation. We were thus able to not only delete but also overactivate myofibroblast YAP and TAZ to study the role of the Hippo pathway in myofibroblasts in driving organ fibrosis. This systematic approach demonstrated that myofibroblast YAP/TAZ and their upstream Hippo kinase regulators are likely critical in regulating fibrogenesis in multiple tissues after injury. Combined with our time course studies demonstrating an early increase in myofibroblast YAP/TAZ activity after tissue injury, our results established myofibroblast Hippo pathway inactivation (and subsequent activation of YAP/TAZ) as an important pathway driving injury-induced organ fibrosis.

In the current study, we knocked out both YAP and TAZ from myofibroblasts, based on findings from our group and others showing that YAP and TAZ play redundant roles in the regulation of fibroblast responses to TGF-β, a critical profibrotic stimulus, as well as fibroblast activation in general (5, 23, 47). Nevertheless, it is possible that YAP or TAZ may play a dominant role in some settings, and so future studies examining the specific effects of YAP or TAZ knockout may be useful. It is important to note, however, that most published data suggest that YAP and TAZ are regulated by the same pathways and mediate their effects primarily through the same TEAD family of transcription factors. As such, specifically targeting YAP or TAZ has proven to be very difficult, and thus most pharmacological strategies under development take a combined YAP/TAZ inhibitory approach.

Although our focus in the current study was on myofibroblast YAP and TAZ, the broader roles that these two proteins play after tissue injury are complex and still being unraveled. Indeed, several studies have shown that in epithelial cells, YAP and/or TAZ are important for proliferative responses after injury, suggesting that YAP/TAZ may play an important role in tissue regeneration, rather than fibrosis (48–50). In contrast, other studies have suggested that epithelial YAP/TAZ activity is important in driving fibrosis (25, 41, 51), at least in part via the secretion of profibrotic molecules that act in a paracrine fashion on neighboring fibroblasts (25). Clearly, YAP and TAZ have multiple and highly nuanced effects, which likely depend upon the cell types being studied, as well as the specific settings of interest. While beyond the scope of the current manuscript, future studies are needed to carefully dissect out the specific roles that YAP and TAZ play not only in different cell types but also in different contexts, such as tissue injury, regeneration, and/or fibrosis.

In summary, using a wide range of mouse and human tissues, we demonstrated that inactivation of the myofibroblast Hippo pathway and subsequent activation of its downstream effectors YAP and TAZ are critical events in driving fibrosis after injury in multiple different organs. Taken together, our data point to myofibroblast YAP/TAZ activation as a potential target for novel antifibrotic therapies, at least in certain settings. Given the importance of fibrosis in the pathogenesis of multiple chronic diseases (1), these findings could have widespread impact.

## Methods

### Human kidney biopsy study 1 (RNA-Seq)

We analyzed data from a convenience sample of 18 archived human transplant kidney biopsy samples stored at St. Michael’s Hospital. All biopsies were reported in a blinded fashion by a renal pathologist according to Banff Criteria. Interstitial fibrosis scores were reported as follows: ci0 (<5% of cortex was fibrotic), ci1 (5%–25% cortex was fibrotic), ci2 (26%–50% cortex was fibrotic), and ci3 (>50% cortex was fibrotic) (52).

#### RNA extraction, cDNA library preparation, and RNA-Seq data processing.

Ten 10 μm thick sections were cut from FFPE kidney biopsy blocks or from CryoMatrix-embedded, unfixed, frozen tissue. RNA was extracted using either a QIAGEN RNeasy FFPE kit (catalog 73504) for FFPE tissue or a QIAGEN Mini Plus kit (catalog 74134) for fresh frozen tissue. Library preparation was performed using commercial kits (Epicentre, EPI-MRZG12324, and Illumina, 15032615 and 15032619), and cDNA libraries were then prepared (Illumina TruSeq RNA sample preparation kit v2, RS-122-2001, or TruSeq Stranded Total RNA Library Prep Kit, RS-122-2301 and RS-122-2302). Libraries were then sequenced using an Illumina HiSeq 2000 or 3000 machine. The resulting RNA-seq data was processed with our in-house pipeline (53). Data were deposited in GEO (accession GSE135327).

### Human kidney biopsy study 2 (analysis of YAP and TAZ nuclear localization)

We analyzed 3 archived FFPE human kidney biopsies stored at St. Michael’s Hospital. The 3 for-cause biopsies were collected serially from the same transplant kidney, with the first obtained at the time of implantation (day 0), the second 9 days after transplant, and the third 2 months after transplant. Interstitial fibrosis was quantified as per the Banff histologic system described above by a blinded pathologist.

### RNA-Seq analyses of published data sets

GSVA (54) was performed in R (v3.6.3) using the GSVA package (v1.34.0, default parameters), using a recently published Yap transcriptional gene set (27) applied to sample data provided in fragments per kilobase of transcript per million mapped reads (FPKM). GSVA scores were then imported into GraphPad Prism (v8.4.3) for further analysis and figure generation. In addition to our own RNA-Seq data derived from our human kidney biopsy study 1, we performed GSVA on RNA-Seq data sets derived in relevant models of fibrotic organ injury published by independent groups as described below.

Bulk RNA-Seq data generated from kidneys obtained at multiple time points after surgery in mice with IRI or sham-operated mice were obtained from GEO (GSE98622) (33). GSVA scores for the YAP transcriptional signature described above were then calculated.

Single-cell RNA-Seq data from mouse lung at baseline and 21 days after bleomycin injury were obtained from GEO (GSE104154) (30). UMI counts of FACS-sorted myofibroblasts [EPCAM^–^CD31^–^CD45^–^TBX4 (TdTomato)^+^ αSMA (GFP)^+^] were extracted using the provided annotations, log-normalized in Seurat using a scale factor of 10,000, and scaled on all genes.

Single-cell RNA-Seq data from mouse liver at baseline and 6 weeks after CCl_4_ injury were obtained from GEO (GSE137720) (31). Raw counts were imported into R (v3.6.3), and barcodes were retained if a minimum of 500 features had nonzero counts and less than 10% of counts were from mitochondrial genes. Data were subsequently analyzed using Seurat (v3.1.5) (55). Similarly, single-cell RNA-Seq data generated from liver tissue of mice with hepatic fibrosis (CCL_4_-induced) or untreated controls were obtained from GEO (GSE132662) (32). Cells with less than 1000 transcripts, less than 500 genes, and a mitochondrial content greater than 15% were excluded. Hepatic stellate cells and myofibroblasts were identified using marker genes supplied in the manuscript, and these populations were subsetted to a smaller object and subjected to GSVA for enriched YAP transcriptional signature genes in fibrotic versus control livers.

Single-cell RNA-Seq data derived from human lung tissue of 20 pulmonary fibrosis and 10 nonfibrotic lungs were obtained from GEO (GSE135893) (28). Using Seurat (v3.2.1), cells with less than 1000 transcripts, less than 1350 genes, and a mitochondrial content greater than 25% were excluded. A smaller object was generated from the 4 identified fibroblast populations and subjected to GSVA for enriched YAP transcriptional signature genes in fibrotic versus control lungs.

In each instance, GSVA was performed on remaining cells using GSVA (v1.34.0, default parameters) as described above. Density plots of GSVA scores for baseline and postinjury myofibroblasts were produced using the R package ggplot2 (v3.3.2), and score distributions were compared by 2-tailed Welch’s *t* test using the base R t.test() function, or the moderated 2-tailed *t* test computed by Limma (56).

### Animal experiments

Mice were kept on a 12-hour light/12-hour dark cycle with ad libitum access to food and water. WT C57BL/6 mice were purchased from Charles River Laboratories (stock no. 027). Two strains of tamoxifen-inducible myofibroblast-specific YAP/TAZ-deficient mice were generated for these studies. *Yap^fl/fl^* *Taz^fl/fl^* mice (on a mixed background) (57) were crossed with C57BL/6J mice containing a tamoxifen-inducible Cre recombinase expressed under the control of either the proα1(1) collagen promoter (Col1a1-Cre/ERT, The Jackson Laboratory, stock no. 016241) or the proα2(I) collagen promoter (Col1a2-Cre/ERT, The Jackson Laboratory, stock no. 029567). Breeding was continued to generate Col1a1-Cre/ERT^+/–^ *Yap^fl/fl^* *Taz^fl/fl^* mice and Col1a2-Cre/ERT^+/–^ *Yap^fl/fl^* *Taz^fl/fl^* mice and their corresponding WT littermate Col1a1-Cre/ERT^–/–^ *Yap^fl/fl^* *Taz^fl/fl^* and Col1a2-Cre/ERT^–/–^ *Yap^fl/fl^* *Taz^fl/fl^* controls. Col1a2-Cre/ERT^+/–^ ROSA26 mTmG^+/–^ *Yap^fl/fl^* *Taz^fl/fl^* mice (*n* = 3) and their WT reporter-labeled controls (Col1a2-Cre/ERT^+/–^ ROSA26 mTmG^+/–^ mice, *n* = 3) were also generated to document myofibroblast-specific YAP knockout. To explore the effects of YAP/TAZ activation, *Lats1^fl/fl^ Lats2^fl/fl^* mice (on a mixed background, a gift of James Martin, Baylor College of Medicine, Houston, Texas, USA) (44) were crossed with mice containing the tamoxifen-inducible Col1a2-Cre/ERT promoter to generate Col1a2-Cre/ERT^+/–^ *Lats1^fl/fl^ Lats2^fl/fl^* mice as well as their WT littermate Col1a2-Cre/ERT^–/–^ *Lats1^fl/fl^ Lats2^fl/fl^* controls.

#### UUO.

Six- to eight-week-old mice underwent sham or left-sided UUO surgery. Briefly, a left-sided flank incision was made in anesthetized mice, and the left kidney and ureter were identified. The left ureter was then obstructed with two 4-0 silk suture ties just distal to the renal pelvis. Seven days after surgery, mice were euthanized and the left kidneys harvested.

#### IRI of the kidney.

C57BL/6 mice (age 6–8 weeks old, *n* = 8) underwent left-sided unilateral IRI surgery as per our previously published protocols (29). During the entire procedure, the core temperature of the mice was maintained between 34°C and 36°C with a heating pad. After induction of anesthesia with inhaled 2% isoflurane, a left-sided flank incision was made, followed by exposure of the pedicle of the left kidney. Hilar vessels were cross-clamped for 45 minutes. Clamps were then removed, allowing the left kidney to reperfuse. Sham-operated mice (*n* = 4) served as healthy controls. Ambient postoperative air temperature was maintained between 30°C and 32°C until mice had fully recovered. Mice were euthanized at various time points after surgery (4 days after IRI, *n* = 4; 14 days after IRI, *n* = 4; 14 days after sham surgery, *n* = 4), and left kidneys were harvested.

#### Bleomycin-induced lung fibrosis.

Twelve-week-old male mice were anesthetized with isoflurane and then received a single intratracheal injection of either saline or bleomycin (0.05 U, Sigma-Aldrich) dissolved in saline. Mice were followed for 14–21 days and then euthanized. Arterial blood oxygenation was measured using a blood gas machine just prior to euthanization (ABL825, Radiometer).

#### CCl_4_-induced liver fibrosis.

Eight-week-old male mice received twice weekly i.p. injections of corn oil or CCl_4_ dissolved in corn oil (1:10 dilution v/v in corn oil, 2 μL/g body weight, Sigma-Aldrich). Mice were followed for 6 weeks and then euthanized.

### Tissue collection, preparation, and histochemistry

At study end, the kidneys, lungs, and/or livers of all mice were harvested. Samples of each kidney were immersion fixed in 10% neutral buffered formalin, embedded in cryostat matrix (Tissue-Tek, VWR), and/or stored in liquid nitrogen. Human and mouse sections were stained with H&E (Leica Biosystems), picrosirius red (Sigma-Aldrich) to label fibrillar collagen, or Masson’s trichrome (Sigma-Aldrich) to stain fibrotic matrix. Ashcroft scoring of lung injury/fibrosis was performed on H&E-stained sections as previously described (42).

### Immunofluorescence staining

FFPE kidney sections were stained with an Alexa Fluor 488–conjugated antibody directed against α-SMA (184675, Abcam) and an antibody directed against YAP (14074, Cell Signaling Technology) or TAZ (83669, Cell Signaling Technology). Primary antibody was detected with CF647-conjugated donkey anti-rabbit IgG (20047, Biotium) followed by nuclear counterstaining with DAPI. Images were taken using a Zeiss LSM 700 inverted laser scanning confocal microscope (58, 59). The percentage of cells with predominantly nuclear YAP or TAZ was calculated as per our previously published protocols (24, 60). Briefly, cells were manually categorized by a blinded observer as having YAP or TAZ localized predominantly in the nucleus, predominantly in the cytoplasm, or neither (24, 60). A minimum of 50 cells per section were examined per replicate using a modified published protocol (60). In some experiments, YAP staining intensity was measured using Fiji (NIH).

### IHC

Formalin-fixed tissues were embedded in paraffin and sectioned before staining with picrosirius red (Sigma-Aldrich) or antibodies against α-SMA (M085129-2, Dako) and type I collagen (1310-01, Southern Biotechnology) (24, 61–63). Four random, nonoverlapping 20× cortical images were taken by a blinded observer using an upright Olympus light microscope, and then analyzed in a blinded fashion using Aperio ImageScope software as previously described (24, 61–63).

### Quantitative reverse-transcription PCR

RNA was collected from mouse kidney and lung tissues. The RNA was then reverse transcribed, and levels of *Col1a1*, *Col3a1*, *Col4a1*, *Acta2*, *Ankrd1*, *Ccn2*, *Ccn1*, *Serpine1*, and/or *Gapdh* were quantified. Primer sequences are summarized in Supplemental Table 1. Experiments were performed in triplicate. Data analyses were performed using the Applied Biosystems Comparative Ct method. All values were referenced to the mRNA transcript levels of the housekeeper gene Gapdh.

### Hydroxyproline content measurement

Hydroxyproline content was measured using a commercial assay kit (K226-110, Biovision), following a slightly modified version of the manufacturer’s protocol (42). Briefly, tissue was homogenized (10 mg per 100 μL water) and hydrolyzed with the addition of 100 μL of 10 N concentrated NaOH to 100 μL of the sample followed by heating at 120°C for 1 hour. After alkaline hydrolysis, the vials were placed on ice to cool. Then, 100 μL of 10 N concentrated HCl was added to neutralize residual NaOH. The samples were then vortexed followed by centrifugation at 10,000*g* for 5 minutes to pellet insoluble debris. Supernatant was then heated at 65°C to enable evaporation, and then chloramine-T concentrate was added to the wells and incubated at room temperature for 20 minutes. To visualize hydroxyproline concentration, 50 μL of developer solution was added to each well and incubated at 37°C for 5 minutes, followed by addition of 50 μL of DMAB concentrate solution and incubation at 65°C for 45 minutes. Sample absorbance was measured at 560 nm using a SpectraMax M5e Multimode plate reader (Molecular Devices). A standard curve was generated using known concentrations of trans-4-hydroxyl-L-proline. Hydroxyproline concentrations from experimental samples were then calculated and normalized to the total amount of soluble protein isolated from 1 mg of liver tissue.

### Atomic force microscopy force spectroscopy

Cell stiffness evaluation was carried out on frozen kidney sections at different time points, using a slightly modified version of our previously published protocol (64). The sections were thawed at room temperature for at least 30 minutes, and then were either fixed with 4% paraformaldehyde for 4 minutes at room temperature or not fixed. In order to identify α-SMA^+^ myofibroblasts, sections were stained with an antibody directed against α-SMA as described above. A single force curve was acquired per interstitial α-SMA^+^ or α-SMA^–^ cell, with a minimum of 15 (for sham animals) and 50 (for UUO animals) randomly chosen cells per section. Every curve was performed by positioning the tip above the cell nucleus. The indentation depth was set at 500 nm in order to measure the cell’s mechanical properties and to reduce the contribution of the underlying surface. The atomic force microscopy measurements were performed using a spherical tip (diameter 20 μm) attached to a silicon nitride cantilever (elastic constant 0.038 N/m and the nominal resonance frequency of 10 kHz, NovaScan). Force-curves were performed at room temperature in the liquid phase using a commercial Bruker Resolve atomic force microscope combined with an inverted optical microscope. Cantilever deflection and z-piezo movements were detected at each indentation step to produce a force-displacement curve by knowing the cantilever spring constant (65). Cell stiffness was determined by fitting the data with a Hertzian model of surface indentation (66) as we performed previously (64).

### Statistics

Statistical analyses of RNA-Seq data sets are described above. A minimum of 3 independent experiments were performed for all in vitro studies. Data presented are mean ± SEM. Between-group differences were measured using a 2-tailed Student’s *t* test or 1-way ANOVA with post hoc Tukey’s analysis where appropriate. Statistical analysis was performed using GraphPad Prism for Mac 6.0. *P* < 0.05 was considered significant.

### Study approval

The human kidney biopsy studies were approved by the St. Michael’s Hospital Research Ethics Board (REB 16-118). The requirement for written informed consent was waived because the study met the criteria for waiver of consent as outlined in the Tri-Council Policy Statement 2 Guidelines (Canada) and Personal Health Information Protection Act (Ontario). All animal studies were approved by the St. Michael’s Hospital Animal Ethics Committee and conformed to the Canadian Council on Animal Care guidelines.

### Data availability

RNA-Seq data are deposited in NCBI’s GEO (GSE135327).

## Author contributions

XH, MFT, SGK, LUS, and SGS designed and performed experiments, conducted data analysis, and revised the manuscript. TZ, PSM, CMM, and LC conducted bioinformatic analyses and revised the manuscript. FG, XC, CA, VK, NV, CH, and JZ performed experiments and analyzed data. CY provided access to and expertise with atomic force microscopy. AK performed human kidney histological analysis. JLW designed experiments, analyzed data, and revised the manuscript. DAY designed experiments, analyzed data, obtained funding, and wrote and revised the manuscript.

## Supplementary Material

Supplemental data

## Version 1 02/22/2022

Electronic publication
