# Supplementary material for: Myofibroblast YAP/TAZ activation is a key step in organ fibrogenesis
Source: JCI Insight. 2022 Feb 22;7(4):e146243. doi: 10.1172/jci.insight.146243 (PMC8876427; doi:10.1172/jci.insight.146243)
Supplement: Supplemental data [file jciinsight-7-146243-s291.pdf]

SUPPLEMENTAL TABLE 1: List of qPCR primer sequences

**Primer**	**Sequence**
Mouse *Col1a1* forward	GAGAACCAGCAGAGCCA
Mouse *Col1a1* reverse	GAACAAGGTGACAGAGGCATA
Mouse *Col3a1* forward	GAAAGGATGGAGAGTCAGGAA
Mouse *Col3a1* reverse	CATTGCGTCCATCAAAGCC
Mouse *Col4a1* forward	TTCTCCCTTTTGTCCCTTCAC
Mouse *Col4a1* reverse	GCTTCTGCTGCTCTTCGC
Mouse *Acta2* forward	CACTGAACCCTAAGGCCAAC
Mouse *Acta2* reverse	GAGTCCAGCACAATACCAGTT
Mouse *Ankrd1* forward	CGA CGT CTG CGA TGA GTA TAA A
Mouse *Ankrd1* reverse	CTC CAG CCT CCA TTA ACT TCT C
Mouse *Ccn2* forward	CCCTAGCTGCCTACCGACT
Mouse *Ccn2* reverse	GGTAACTCGGGTGGAGATGC
Mouse *Ccn1* forward	GAA GGC AGA CCC TGT GAA TAT AA
Mouse *Ccn1* reverse	ACG GCG CCA TCA ATA CAT
Mouse *Serpine1* forward	CGT GTC AGC CTC GTC TAC AG
Mouse *Serpine1* reverse	CTA TGG TGA AAC AGG TGG ACT
Mouse *Gapdh* forward	CACCATCCGGGTTCCTATAAAT
Mouse *Gapdh* reverse	TGGCACTGCACAAGAAGAT

SUPPLEMENTAL FIGURES

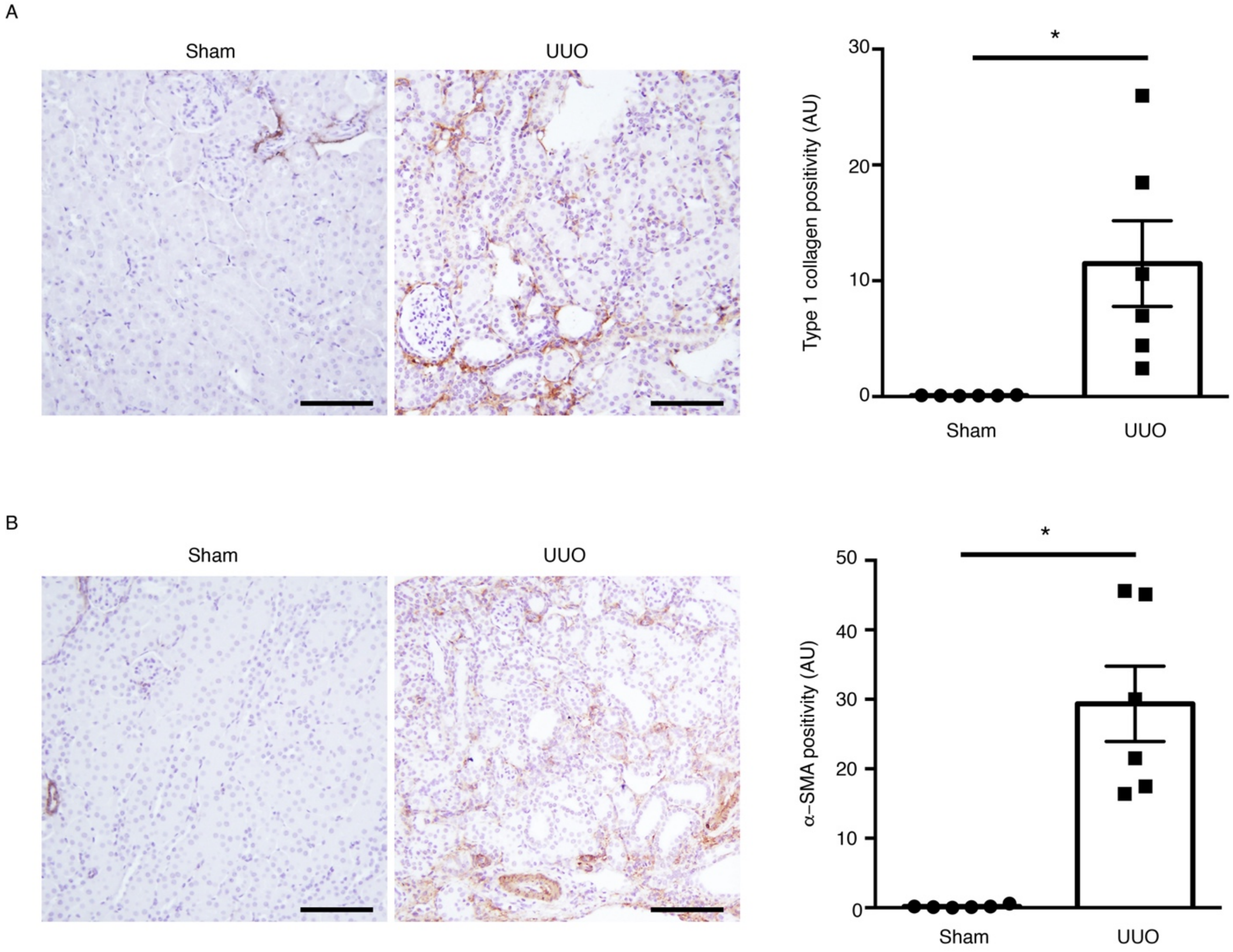

Figure S1: Renal fibrosis parameters in sham and unilateral ureteral obstruction (UUO) mice. Male C57BL/6 mice underwent sham (n = 6) or left-sided UUO surgery (n = 6). Kidney sections from mice 7 days post-surgery were stained with antibodies directed against: (A) type 1 collagen and (B) alpha-smooth muscle actin (α-SMA). One-way ANOVA with post-hoc Fisher's least significant difference was used for comparisons. Scale bar: 100 μm. Error bars denote standard error of mean. * $p < 0.05$. Abbreviations: AU, arbitrary units.

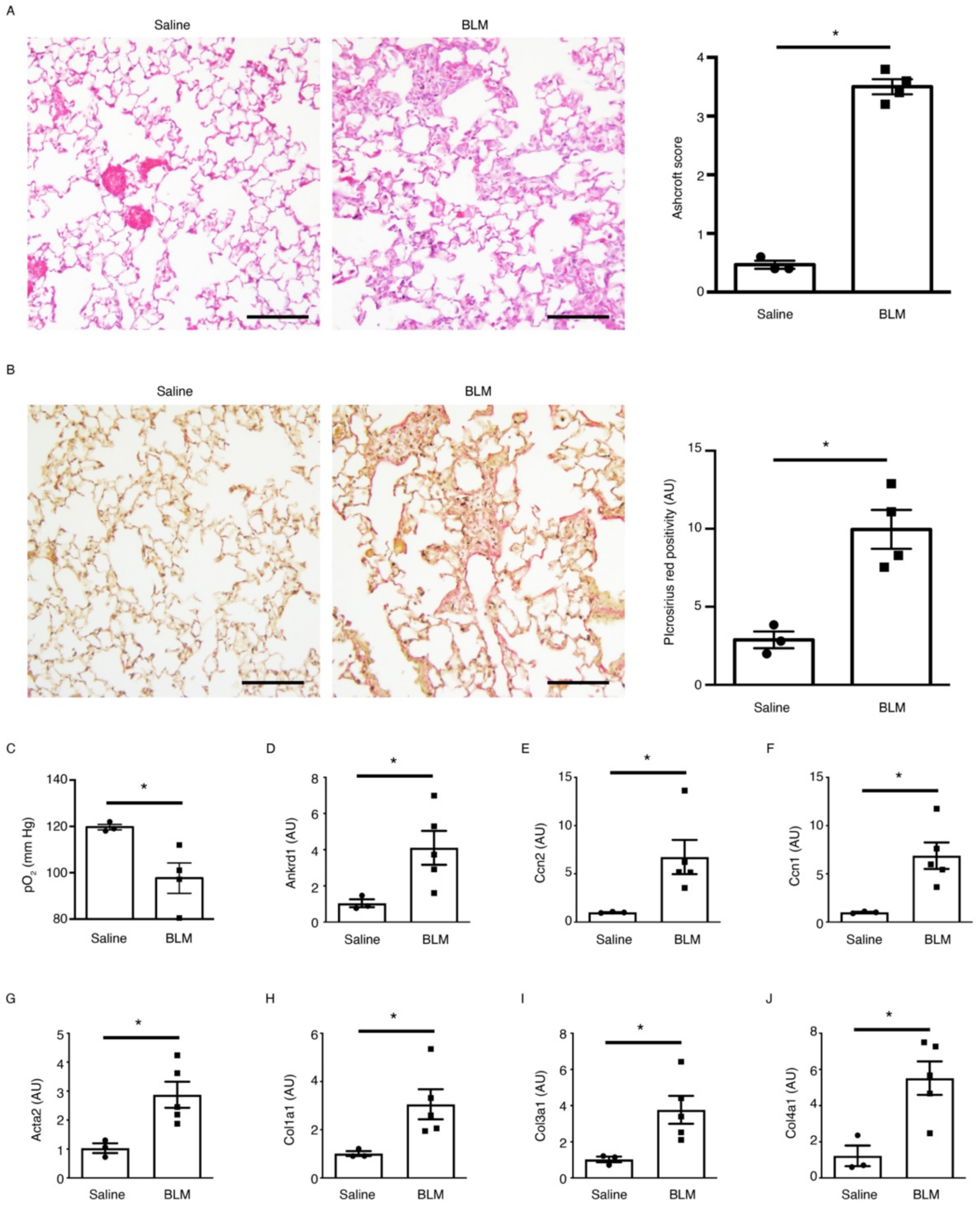

Figure S2: Lung fibrosis parameters in mice administered saline or bleomycin (BLM).
Male C57BL/6 mice received a single intratracheal injection of saline (n = 3) or bleomycin (n = 5). Lung sections from mice 14 days post-injection were stained with: (A) hematoxylin and eosin (H+E) and (B) picrosirius red (to quantify fibrotic injury). (C) Arterial blood oxygenation was measured just prior to sacrifice. (D – J) The mRNA levels of the YAP/TAZ-inducible genes (D) *Ankrd1*, (E) *Ccn2*, and (F) *Ccn1*, and the fibrosis-associated genes (G) *Acta2*, (H) *Col1a1*, (I) *Col3a1*, and (J) *Col4a1* were examined using qPCR of cDNA prepared from whole lung homogenates. Transcript levels were normalized to the housekeeper transcript *Gapdh*. Student's t-test was used for comparisons. Scale bar: 100 μm. Error bars denote standard error of mean. * $p \leq 0.05$. Abbreviations: AU, arbitrary units.

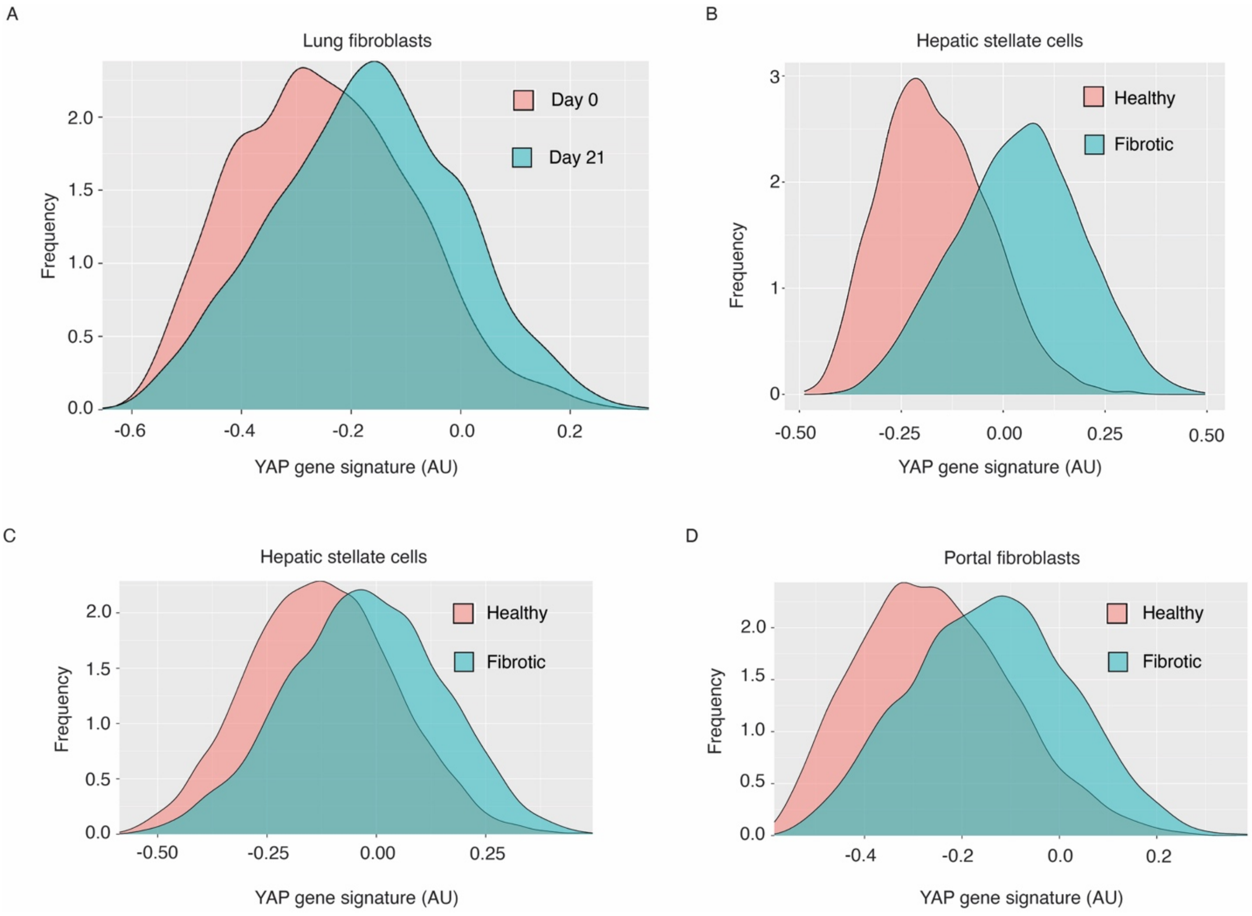

Figure S3: Fibrotic injury activates a YAP-driven gene program in multiple mouse organs. YAP-driven transcription was examined in several publicly available RNA-seq datasets. (A) An increase in a YAP transcriptional signature [27] was noted in fibroblasts isolated from murine lungs 21 days post-bleomycin administration (n = 10), as compared with fibroblasts isolated from lungs prior to bleomycin administration [30]. Adjusted $p < 2.2 \times 10^{-16}$. (B) This same YAP transcriptional signature was increased in (B – C) hepatic stellate cells and (D) portal fibroblasts isolated from carbon tetrachloride-induced fibrotic mouse livers, when compared with their counterparts isolated from healthy mouse liver controls [31,32]. Adjusted $p < 2.2 \times 10^{-16}$ for all comparisons in the liver. Student's t test with Welch's correction or the moderated t test computed by Limma was used for comparisons. Error bars denote standard error of mean. Abbreviations: AU, arbitrary units. FPKM, fragments per kilobase million.

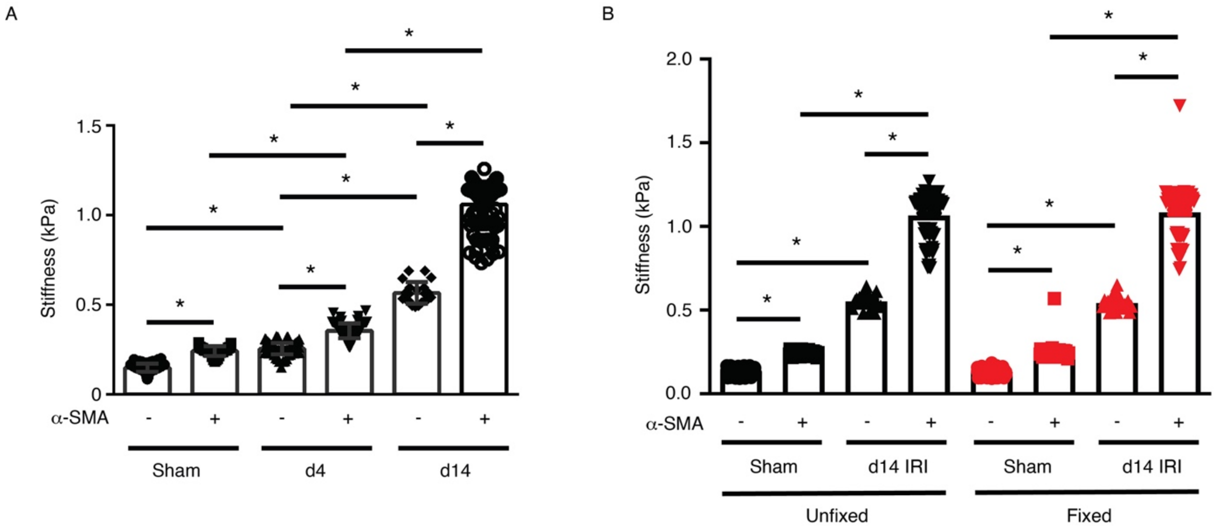

Figure S4: Interstitial cells, including α-SMA$^+$ myofibroblasts, stiffen over time following renal ischemia-reperfusion injury.

Male C57BL/6 mice underwent sham (n = 4) or left-sided unilateral ischemia-reperfusion injury (IRI, n = 8) surgery. IRI mice were sacrificed at early (4 days, n = 4) or late (14 days, n = 4) time points post-injury. Sham-operated mice were sacrificed at 14 days post-surgery. The stiffness of interstitial cells, including both α-smooth muscle actin$^+$ (α-SMA$^+$) myofibroblasts and α-SMA$^-$ cells, was quantified by atomic force microscopy using kidney sections that were briefly fixed with paraformaldehyde (PFA) for 4 minutes. (A) Mean α-SMA$^+$ myofibroblast and α-SMA$^-$ cell stiffness in kidneys from mice 4 and 14 days post-IRI, as well as from sham-operated healthy controls. (B) To determine whether stiffness was affected by brief PFA fixation, experiments were repeated using unfixed kidney sections from sham and 14 day post-IRI animals. As shown in (A) and (B), stiffness of both α-SMA$^+$ myofibroblasts and α-SMA$^-$ cells increased with time post-IRI, but were unaffected by PFA fixation. Each dot in the graphs represents an individual cell. One-way ANOVA with post-hoc Tukey's test was used for comparisons. Error bars denote standard error of mean. * $p < 0.05$. Abbreviations: d, day. kPa, kiloPascals.

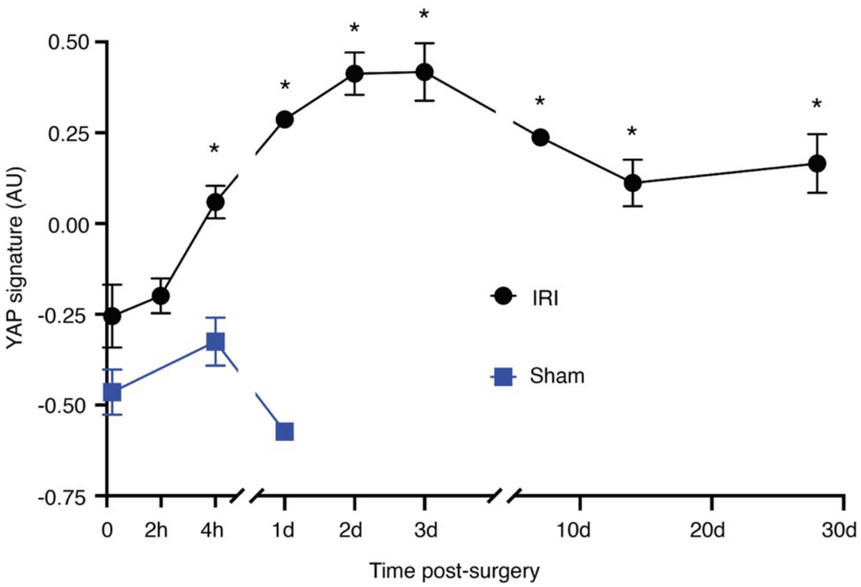

Figure S5: YAP transcriptional signature is increased following ischemia-reperfusion injury (IRI) in mouse kidneys.

A published bulk RNA-seq dataset derived from mouse kidney samples collected at various times post-ischemia-reperfusion injury or sham surgery was analyzed [33]. A YAP transcriptional signature was analyzed by gene set variation analysis. One-way ANOVA with post-hoc Tukey's test was used for comparisons. Error bars denote standard error of mean. * $p < 0.05$ when compared to sham animals at the first time point (3 minutes post-surgery). Abbreviations: AU, arbitrary units. d, days. h, hours.

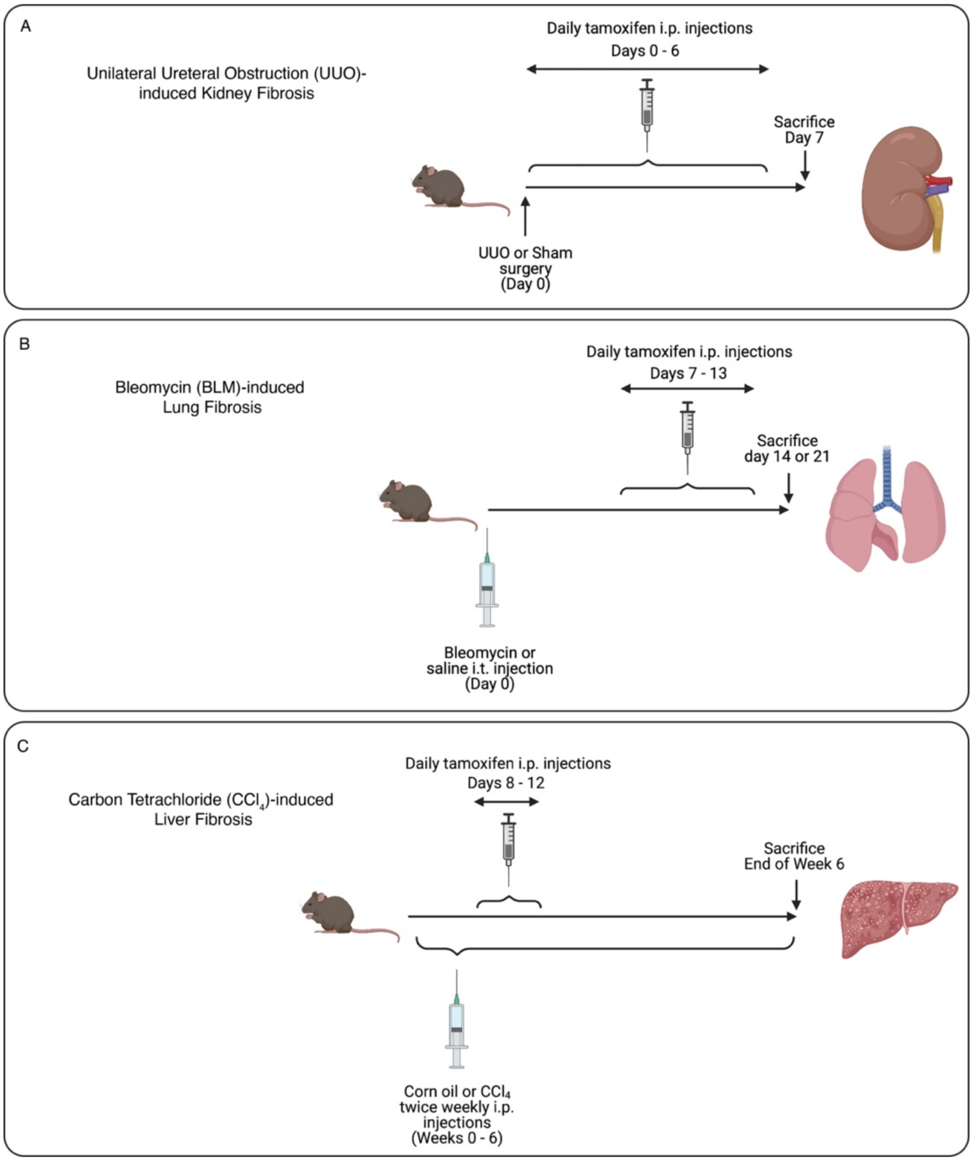

Figure S6: Experimental design for fibroblast-specific deletion of *Yap*/*Taz* or *Lats1/2* in models of murine kidney, lung, and/or liver fibrosis.

Mice expressing a tamoxifen-inducible collagen promoter-driven Cre recombinase (Col1a1-Cre/ERT$^{+/-}$ or Col1a2-Cre/ERT$^{+/-}$), as well as either floxed *Yap* and *Taz* (*Yap*$^{fl/fl}$, *Taz*$^{fl/fl}$) or floxed *Lats1* and *Lats2* (*Lats1*$^{fl/fl}$, *Lats2*$^{fl/fl}$), were subjected to (A) unilateral ureteral obstruction (UUO)-induced kidney fibrosis, (B) bleomycin (BLM)-induced lung fibrosis, or (C) carbon tetrachloride (CCl_4)-induced liver fibrosis. Details of each experiment are provided in the Methods section and in the corresponding Figure legends. Abbreviations: i.p., intra-peritoneal. i.t., intra-tracheal. Created using BioRender.com.

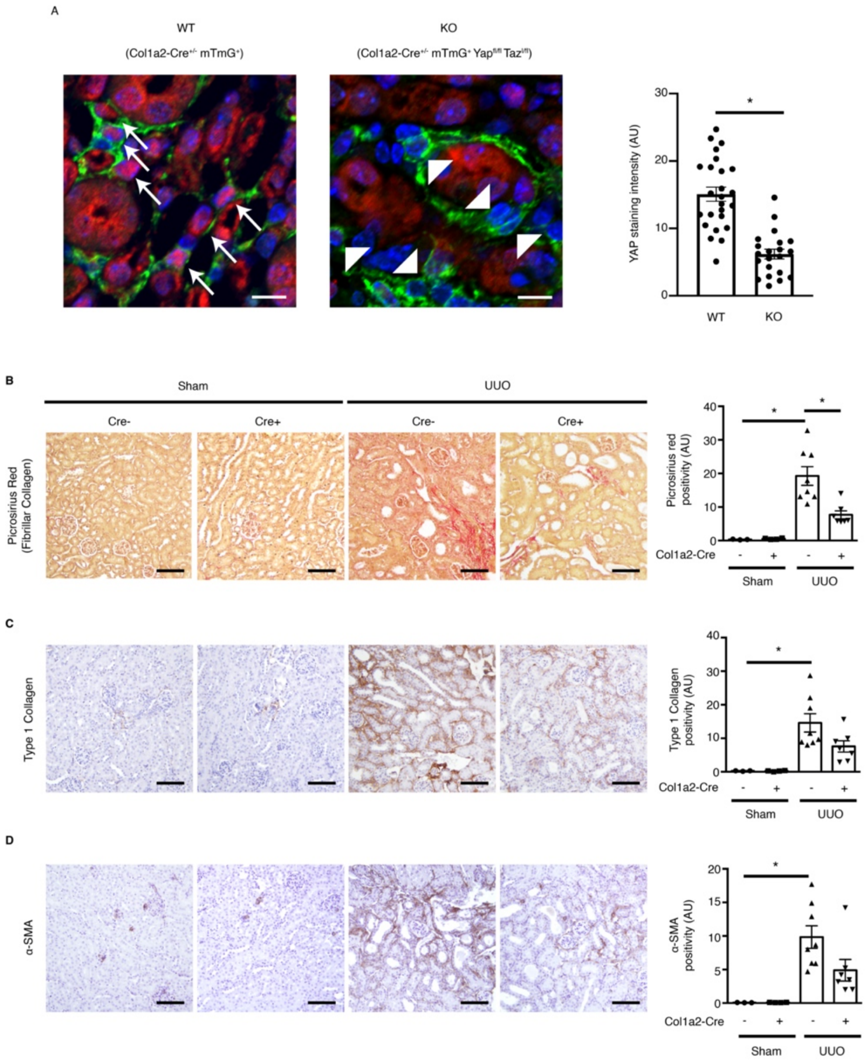

Figure S7: Col1a2-Cre/ERT$^{+/-}$ *Yap*$^{fl/fl}$ *Taz*$^{fl/fl}$ mice are protected against unilateral ureteral obstruction (UUO)-induced kidney fibrosis. Fibroblast-specific YAP/TAZ deficient mice expressing an mTmG reporter (Col1a2-Cre/ERT$^{+/-}$ ROSA$^{mT/mG}$ *Yap*$^{fl/fl}$ *Taz*$^{fl/fl}$) and their wild type littermates (Col1a2-Cre/ERT$^{+/-}$ ROSA$^{mT/mG}$) underwent left-sided unilateral ureteral obstruction (UUO, n = 3/group). Tamoxifen was administered between days 0 and 6 post-surgery to activate expressed Cre recombinase. The mTmG reporter expresses membrane-targeted tdTomato in all cells, except those with active Cre recombinase, in which case the tdTomato cassette is deleted and a membrane-targeted EGFP protein is expressed. Left kidneys were harvested 7 days post-surgery. EGFP staining marks cells expressing active Cre recombinase. (A) To document successful knockout, sections were stained with antibodies directed against EGFP (green) and YAP (red), with DAPI nuclear counterstaining. White arrows depict EGFP^{+} myofibroblasts with robust nuclear YAP expression in wild type mice. White arrowheads identify EGFP^{+} myofibroblasts with no YAP staining, indicating successful YAP deletion. White scale bar: 10 μm. YAP staining intensity in EGFP^{+} myofibroblasts was quantified. Each data point in the graph represents an individual image (n = 25 WT, n = 21 KO). Additional Col1a2-Cre/ERT$^{+/-}$ Yap$^{fl/fl}$ Taz$^{fl/fl}$ mice and their wild type Col1a2-Cre/ERT$^{-/-}$ *Yap*$^{fl/fl}$ *Taz*$^{fl/fl}$ littermates underwent sham surgery (n = 3, Col1a2-Cre/ERT$^{-/-}$ *Yap*$^{fl/fl}$ *Taz*$^{fl/fl}$ and n = 4, Col1a2-Cre/ERT$^{+/-}$ *Yap*$^{fl/fl}$ *Taz*$^{fl/fl}$) or UUO surgery (n = 8, Col1a2-Cre/ERT$^{-/-}$ *Yap*$^{fl/fl}$ *Taz*$^{fl/fl}$ and n = 7, Col1a2-Cre/ERT$^{+/-}$ *Yap*$^{fl/fl}$ *Taz*$^{fl/fl}$). Kidney sections were stained with (B) picrosirius red (PSR) to label fibrillar collagen, (C) an antibody against type 1 collagen, or (D) an antibody against α-smooth muscle actin (α-SMA). Black scale bar: 100 μm. Two-tailed Student's t test or one-way ANOVA with post-hoc Tukey's test was used for comparisons. Error bars denote standard error of mean. * $p < 0.05$. Abbreviations: AU, arbitrary units. KO, knockout. WT, wild type.

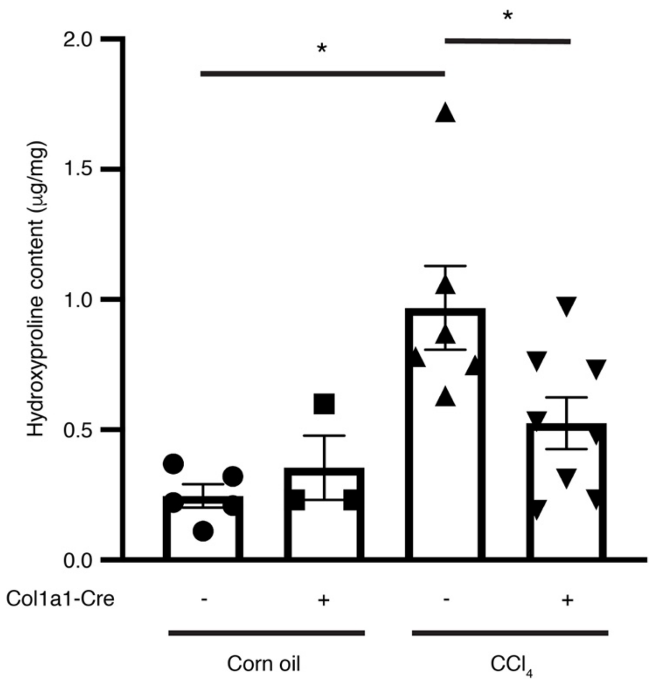

Figure S8: Col1a1-Cre/ERT$^{+/-}$ *Yap*$^{fl/fl}$ *Taz*$^{fl/fl}$ mice have reduced hepatic hydroxyproline content, a measure of liver fibrosis, following carbon tetrachloride (CCl_4)-induced liver injury. Myofibroblast-specific YAP/TAZ-deficient mice (Col1a1-Cre/ERT$^{+/-}$ *Yap*$^{fl/fl}$ *Taz*$^{fl/fl}$) and their wild type littermates (Col1a1-Cre/ERT$^{-/-}$ *Yap*$^{fl/fl}$ *Taz*$^{fl/fl}$) were randomized to corn oil (n = 5 wild type littermates, n = 3 myofibroblast-specific YAP/TAZ-deficient mice) or carbon tetrachloride injections (n = 6 wild type littermates, n = 8 myofibroblast-specific YAP/TAZ-deficient mice), and tamoxifen was administered between days 8 – 12 after the initiation of corn oil/CCl_4 injections. Liver samples were flash frozen in liquid nitrogen 6 weeks after the first corn oil/CCl_4 injection, and hydroxyproline content was measured (expressed as μg per mg of soluble protein) in a subset of mice. One-way ANOVA with post-hoc Tukey's test was used for comparisons. Error bars denote standard error of mean. * $p < 0.05$.

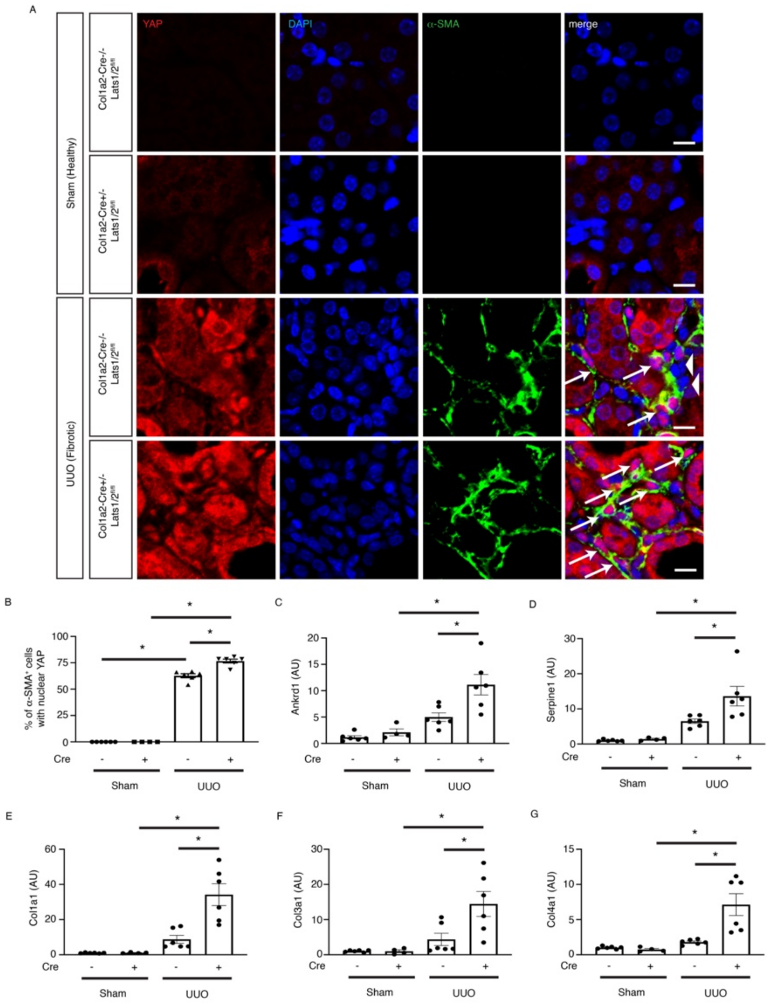

Figure S9: Myofibroblast-specific LATS1/2 deficiency is associated with YAP hyperactivation in fibroblasts. Myofibroblast-specific LATS1/2-deficient mice (Col1a2-Cre/ERT$^{+/-}$ *Lats1*$^{fl/fl}$ *Lats2*$^{fl/fl}$) and their wild type littermates (Col1a2-Cre/ERT$^{-/-}$ *Lats1*$^{fl/fl}$ *Lats2*$^{fl/fl}$) underwent left-sided unilateral ureteral obstruction (UUO, n = 6/group) or sham surgery (n = 4 fibroblast-specific LATS1/2-deficient mice, n = 6 wild type littermates). Tamoxifen was administered between days 0 and 6 post-surgery to activate expressed Cre recombinase. Left kidneys were harvested 7 days post-surgery. (A – B) To document successful knockout, kidney sections were stained with antibodies directed against alpha-smooth muscle actin (α-SMA) and YAP, with DAPI nuclear counterstaining. As shown in the lower panels, myofibroblast-specific LATS1/2-deficient UUO mice (fourth row) had greater numbers of α-SMA^{+} myofibroblasts with nuclear YAP compared to wild type UUO mice (third row). White arrows depict α-SMA^{+} myofibroblasts with nuclear YAP expression. White arrowheads depict α-SMA^{+} fibroblasts with non-nuclear YAP expression. Scale bar: 10 μm. The expression of the YAP/TAZ-inducible genes (C) *Ankrd1* and (D) *Serpine1*, as well as the collagens (E) *Col1a1*, (F) *Col3a1*, and (G) *Col4a1* were measured by qPCR, relative to that of Gapdh (a housekeeper control). One-way ANOVA with post-hoc Tukey's test was used for comparisons. Error bars denote standard error of mean. * $p < 0.05$. Abbreviations: AU, arbitrary units.

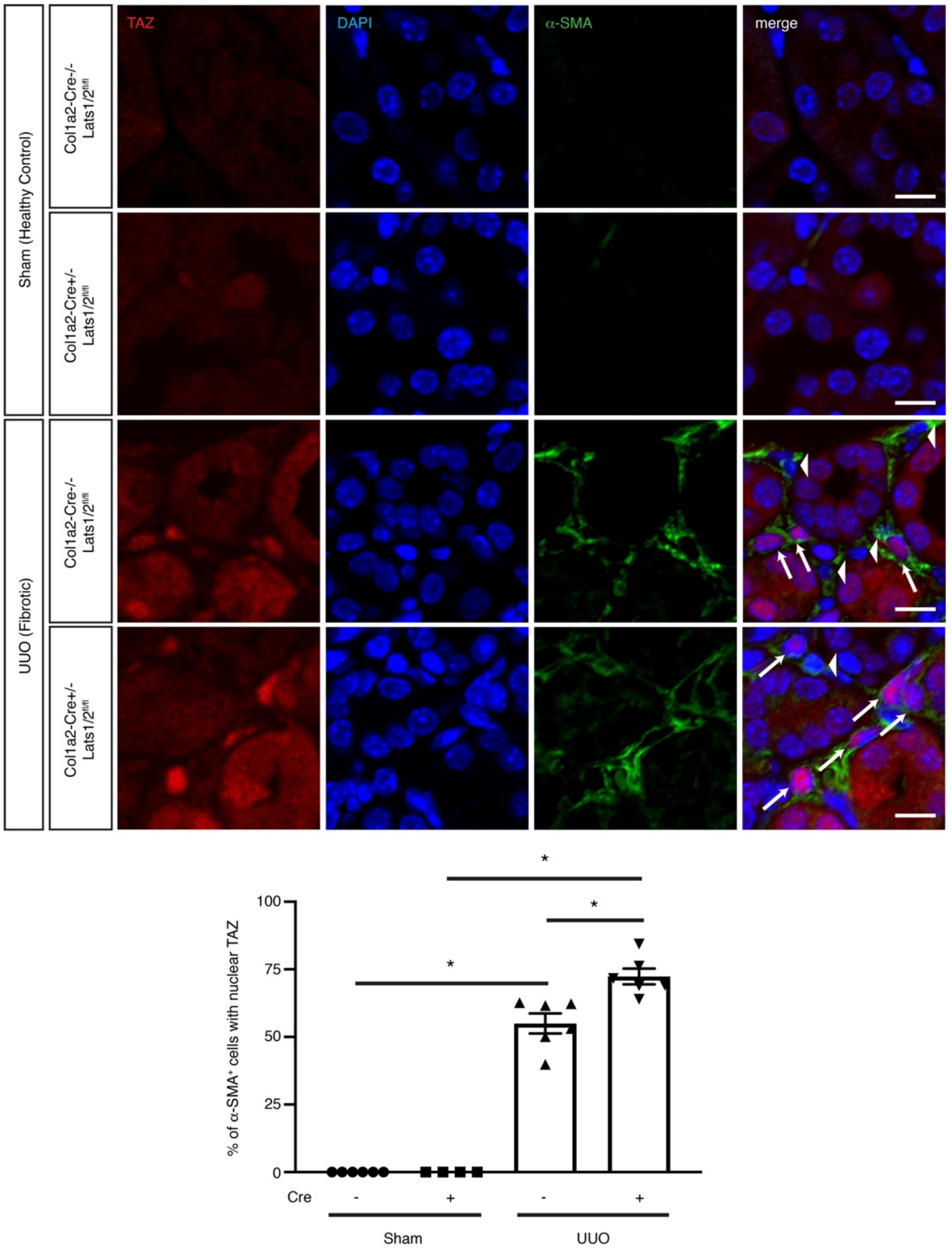

Figure S10: Myofibroblast-specific LATS1/2 deficiency is associated with TAZ hyperactivation in fibroblasts. Kidney sections from the myofibroblast-specific LATS1/2-deficient mice (Col1a2-Cre/ERT$^{+/-}$ *Lats1*$^{fl/fl}$ *Lats2*$^{fl/fl}$, n = 4 sham, n = 6 UUO) and wild type control mice (Col1a2-Cre/ERT$^{-/-}$ *Lats1*$^{fl/fl}$ *Lats2*$^{fl/fl}$, n = 6 sham, n = 6 UUO) described in Figure S9 were stained with antibodies directed against alpha-smooth muscle actin (α-SMA) and TAZ, with DAPI nuclear counterstaining. White arrows depict α-SMA$^+$ myofibroblasts with nuclear TAZ expression. White arrowheads depict α-SMA$^+$ myofibroblasts with non-nuclear TAZ expression. As shown in the lower panels, myofibroblast-specific LATS1/2-deficient UUO mice (Col1a2-Cre/ERT$^{+/-}$ *Lats1*$^{fl/fl}$ *Lats2*$^{fl/fl}$, fourth row) demonstrated more frequent α-SMA$^+$ myofibroblasts with nuclear TAZ, as compared with their wild type littermate UUO controls (third row). Scale bar: 10 μm. One-way ANOVA with post-hoc Tukey's test was used for comparisons. Error bars denote standard error of mean. Abbreviations: UUO, unilateral ureteral obstruction.
